# Photoimmunotherapy Meets Systemic Immunotherapy: Molecular Mechanisms, Microenvironmental Remodeling, and Combinatorial Paradigms in Solid Tumors

**DOI:** 10.3390/ijms27219854

**Published:** 2026-11-03

**Authors:** Francesco Russano, Francesco Callegarin, Davide Brugnolo, Marco Rastrelli, Rocco Caminiti, Giuseppe Sciacca, Daniele Scafuri, Marcodomenico Mazza, Paolo Del Fiore, Luigi Dall’Olmo, Simone Mocellin

**Affiliations:** 1 Soft-Tissue, Peritoneum and Melanoma Surgical Oncology Unit, Veneto Institute of Oncology IOV—IRCCS, 35128 Padua, Italy; francesco.russano@iov.veneto.it (F.R.); marco.rastrelli@unipd.it (M.R.); marcodomenico.mazza@iov.veneto.it (M.M.); paolo.delfiore@iov.veneto.it (P.D.F.); luigi.dallolmo@unipd.it (L.D.); simone.mocellin@unipd.it (S.M.); 2 Clinical Research Unit, Veneto Institute of Oncology IOV—IRCCS, 35128 Padua, Italy; 3 Department of Surgery, Oncology and Gastroenterology (DISCOG), University of Padua, 35020 Padua, Italy; davide.brugnolo@iov.veneto.it (D.B.); giuseppe.sciacca@studenti.unipd.it (G.S.); daniele.scafuri@studenti.unipd.it (D.S.); 4 Casa di Cura Caminiti, Villa San Giovanni, 89018 Reggio Calabria, Italy; rocco.caminiti@icloud.it

**Keywords:** photoimmunotherapy, NIR-PIT, immunogenic cell death, tumor microenvironment, cGAS-STING pathway, immune checkpoint blockade, combination immunotherapy, solid tumors

## Abstract

Photodynamic therapy (PDT) remains constrained by physical barriers, including strict oxygen dependence, acute hypoxia exacerbation, and limited light penetration depth. While conventional photodynamic therapy (PDT) is strictly oxygen-dependent and limited by light scattering, Near-Infrared Photoimmunotherapy (NIR-PIT) overcomes microenvironmental hypoxia through targeted, oxygen-independent biophysical membrane rupture. However, tissue optical attenuation at 690 nm remains a fundamental physical boundary that necessitates interstitial fiber placement for deep lesions. Concurrently, recent advances in bioengineered nano-photoimmunotherapy (nano-PIT) and organelle-targeted photodynamic/photothermal platforms deliberately execute alternative inflammatory non-apoptotic pathways, such as pyroptosis, cuproptosis, and ferroptosis, and provoke cytosolic leakage of mitochondrial DNA that engages the cGAS-STING innate immune axis. Concurrently, NIR-PIT comprehensively remodels the immunosuppressive tumor microenvironment (TME) by selectively depleting regulatory T cells (Tregs), myeloid-derived suppressor (MDSCs) cells, and cancer-associated fibroblasts (CAFs), thereby deconstructing desmoplastic stroma, lowering interstitial fluid pressure (IFP), and driving the Super-Enhanced Permeability and Retention (SUPR) effect. Photically released mitochondrial DNA further engages the cytosolic cGAS-STING pathway, stimulating type I interferon signaling and dendritic cell cross-presentation. In selected preclinical models, this biophysical collapse promotes immunogenic cell death (ICD) and tumor microenvironment remodeling. When combined with systemic immunotherapies, NIR-PIT has demonstrated the potential to prime antitumor immunity and elicit abscopal responses in responsive syngeneic models; however, this immune conversion is not universal and is heavily constrained by tumor mutational burden, baseline antigen presentation fidelity, and diverse immunosuppressive networks. Translating these paradigms into clinical practice requires advancing 3D microphysiological models, implantable light technologies, and multi-component dosimetry frameworks.

## 1. Introduction

**Figure 1:**
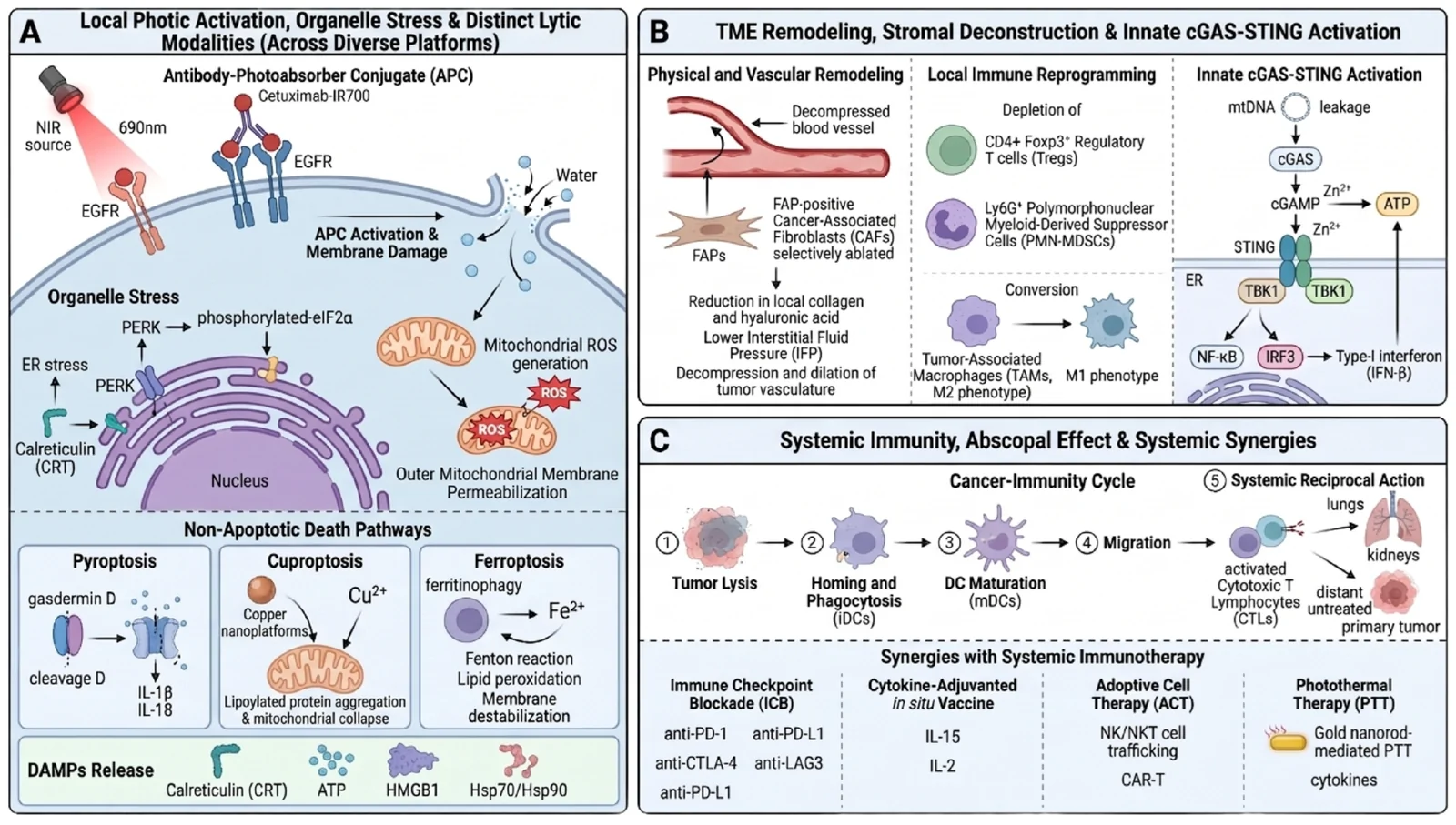
Mechanistic landscape, microenvironmental remodeling, and systemic immunotherapeutic synergies across phototherapeutic platforms. (**A**) Local photic activation, organelle stress, and non-apoptotic lytic cascades: The panel provides a comparative conceptual overview of distinct cytotoxic modalities established across different light-activated technologies, rather than a single unified cascade. Irradiation with 690 nm NIR light activates canonical antibody-photoabsorber conjugates (APC, e.g., cetuximab-IR700) bound to EGFR, inducing rapid, oxygen-independent biophysical membrane rupture and osmotic water influx. Concurrently, specialized bioengineered nanomedicines and organelle-targeted photosensitizers induce focal organelle stress (ER stress via the Protein Kinase RNA-like Endoplasmic Reticulum Kinase (PERK)–phosphorylated-eIF2α pathway and outer mitochondrial membrane permeabilization) or execute distinct, non-apoptotic lytic cascades: gasdermin D cleavage in pyroptosis with IL-1β/IL-18 release; copper nanoplatform-mediated Cu^2+^ accumulation driving toxic lipoylated protein aggregation and mitochondrial collapse in cuproptosis; and ferritinophagy-driven lipid peroxidation in ferroptosis. These pathways drive the coordinated emission of damage-associated molecular patterns (DAMPs). (**B**) TME remodeling, stromal deconstruction, and innate cyclic GMP-AMP synthase (cGAS)—stimulator of interferon genes (STING) ativation: Physical and Vascular Remodeling: Selective ablation of FAP-positive cancer-associated fibroblasts (CAFs) leads to a reduction in local collagen and hyaluronic acid, lowers interstitial fluid pressure (IFP), and promotes the decompression and dilation of tumor blood vessels. Local Immune Reprogramming: Photic depletion of CD4^+^ Foxp3^+^ regulatory T cells (Tregs) and Ly6G^+^ polymorphonuclear myeloid-derived suppressor cells (PMN-MDSCs) facilitates the conversion of immunosuppressive M2-like tumor-associated macrophages (TAMs) into a pro-inflammatory M1-like phenotype along the activation continuum. Innate cGAS-STING Activation: Photically induced mitochondrial stress causes cytosolic leakage of double-stranded mitochondrial DNA (mtDNA), activating cGAS to synthesize 2′,3′-cGAMP (amplified by Zn^2+^). cGAMP binds STING on the ER membrane, recruiting TBK1 to trigger NF-κB and IRF3 activation and drive Type-I interferon (IFN-β) expression, while extracellular ATP serves as a homing beacon for dendritic cells. (**C**) Systemic immunity, abscopal effect, and systemic synergies: The cancer-immunity cycle proceeds via (1) primary tumor lysis, (2) homing and antigen phagocytosis by immature dendritic cells (iDCs), (3) DC maturation (mDCs), (4) migration to draining lymph nodes for CD8^+^/CD4^+^ T-cell activation, and (5) systemic reciprocal action of activated cytotoxic T lymphocytes (CTLs). CTLs circulate to primary and distant untreated tumors as well as visceral organ sites (e.g., lungs, kidneys), mediating the systemic abscopal effect. These photic mechanisms synergize with systemic immunotherapeutic modalities, including immune checkpoint blockade (ICB: anti-PD-1, anti-PD-L1, anti-CTLA-4, anti-LAG3), cytokine-adjuvanted in situ vaccines (IL-15, IL-2), adoptive cell therapies (ACT: NK/NKT cell trafficking, CAR-T), and photothermal therapy (PTT: gold nanorod-mediated PTT with cytokines). Created in BioRender. Callegarin, F. (2026) https://BioRender.com/aidq49i (accessed on 29 October 2026).

Conventional photodynamic therapy (PDT) is a clinically established, minimally invasive modality that generates cytotoxic reactive oxygen species (ROS) to ablate tumors. It relies on the simultaneous presence of a non-toxic photosensitizer (PS), a specific wavelength of light, and molecular oxygen. Upon light absorption, the PS is excited to a short-lived singlet state and undergoes intersystem crossing to a triplet state. Through Type-I photochemical pathways, it generates free radicals, whereas through Type-II pathways, it transfers energy directly to ground-state molecular oxygen to produce highly reactive singlet oxygen (^1^O_2_). This transient singlet oxygen induces localized damage to lipids, proteins, and tumor vasculature, culminating in cell death [1,2]. Despite its clinical utility in treating superficial malignancies, conventional PDT is fundamentally constrained by physical barriers. First, because most approved PSs rely heavily on the Type-II singlet oxygen pathway, their efficacy is strictly oxygen-dependent. Second, the vis-light wavelengths commonly used (typically 400 to 700 nm) suffer from severe scattering and absorption by endogenous tissue chromophores such as hemoglobin and water. This restricts light penetration to less than 5 mm, rendering deep-seated, bulky, or metastatic tumors entirely untreatable [2,3]. The hypoxic tumor microenvironment (TME) of solid tumors represents a major physiological obstacle. Pre-existing hypoxia (pO2 < 10 mmHg) severely blunts the generation of singlet oxygen. More critically, Type-II PDT is inherently self-limiting: the rapid, photodynamically driven consumption of oxygen outpaces local blood supply, acutely exacerbating intratumoral hypoxia. This depletion of oxygen triggers the stabilization of Hypoxia-Inducible Factor 1-alpha (HIF-1α). HIF-1α stabilization upregulates pro-angiogenic factors (such as Vascular Endothelial Growth Factor, VEGF) and shifts cell metabolism toward glycolysis, accelerating tumor survival and recurrence [4]. Simultaneously, this exacerbated hypoxia fosters a highly immunosuppressive niche. The up-regulation of immune checkpoint ligands (such as Programmed Death-Ligand 1 [PD-L1]) on tumor and myeloid cells actively silences infiltrating cytotoxic T lymphocytes (CTLs). Consequently, PDT conducted as a monotreatment fails to elicit a protective systemic antitumor immune response, often leading to compensatory immune evasion and unchecked metastatic outgrowth [3,5]. To circumvent the oxygen-dependence of conventional PDT, Near-Infrared Photoimmunotherapy (NIR-PIT, Alluminox™) has emerged as a targeted biophysical modality. NIR-PIT utilizes an antibody-photoabsorber conjugate (APC) composed of a hydrophilic silicon-phthalocyanine dye (IRDye700DX, IR700) covalently bound to a humanized monoclonal antibody targeting specific tumor receptors, such as Epidermal Growth Factor Receptor (EGFR) or CD44. The benchmark APC, cetuximab sarotalocan, is clinically approved in Japan for unresectable, locally recurrent head and neck squamous cell carcinoma (HNSCC) [6,7]. Unlike conventional PDT, the primary cytotoxic mechanism of NIR-PIT is entirely oxygen-independent: upon exposure to 690 nm NIR light, photochemical cleavage of the axial ligands triggers a rapid hydrophilic-to-hydrophobic phase transition, receptor aggregation, and physical membrane rupture resulting in osmotic lysis within minutes [8,9]. Nevertheless, it must be emphasized that NIR-PIT does not circumvent the fundamental physics of photon attenuation in human tissue [10]. Although 690 nm light falls within the near-infrared biological window, photon scattering and tissue absorption limit direct non-invasive surface penetration to approximately 5–10 mm, requiring invasive cylindrical diffusing fibers or endoscopic delivery for tumors exceeding this depth [10]. Furthermore, while the sudden oncosis promotes the release of antigens and endogenous adjuvants, turning treated sites into inflamed foci, the extent of subsequent immune engagement varies considerably according to host immune status and tumor biology [11,12]. Consequently, a highly localized biophysical treatment is transformed into a systemic ‘in situ’ vaccine [11,13]. Furthermore, the rapid clearance of perivascular cancer cells induces a massive increase in local vascular permeability, known as the Super-Enhanced Permeability and Retention (SUPR) effect. The SUPR effect dramatically drops local IFP, enabling up to a 24-fold increase in the intratumoral delivery of subsequently administered systemic therapies, including nanocarriers and immune checkpoint inhibitors. By integrating physical debulking with the recruitment of systemic adaptive immunity, NIR-PIT effectively converts immunologically ‘cold’ tumors into ‘hot’ tumors, laying the groundwork for synergies with systemic immunotherapies discussed in later chapters (Figure 1) [6,11].

### Scope and Mechanistic Demarcation of Phototherapeutic Modalities

To establish clear conceptual boundaries and resolve ambiguities regarding light-mediated immune effects, light-activated oncological modalities reviewed herein must be systematically categorized into three distinct biological and therapeutic paradigms:

Classical Antibody-IR700 NIR-PIT (Alluminox™ platform): A strictly targeted molecular modality utilizing an antibody-photoabsorber conjugate (APC) composed of a monoclonal antibody (e.g., cetuximab) covalently linked to the silica-phthalocyanine dye IRDye700DX [8]. Activated exclusively by 690 nm light, its cytotoxic mechanism is predominantly oxygen-independent [8]. Irradiation triggers the photochemical release of hydrophilic axial ligands, inducing a rapid hydrophilic-to-hydrophobic phase transition. This causes targeted cell-surface receptor aggregation, severe shear stress on the lipid bilayer, and rapid biophysical membrane rupture leading to osmotic lysis within minutes [8]. Importantly, NIR-PIT encompasses two operational approaches depending on the cellular target: (i) Cancer-targeted NIR-PIT (e.g., cetuximab-IR700), where light-induced oncosis releases native antigens and DAMPs to secondarily prime host immunity; and (ii) Direct immune-targeted NIR-PIT (e.g., anti-CTLA-4-IR700, anti-CD25-IR700), where light directly and selectively destroys intratumoral immunosuppressive cells (such as Tregs) to immediately unleash pre-existing antitumor CTLs without requiring tumor cell destruction [14,15,16].

Conventional Photodynamic Therapy (PDT): A photochemical modality requiring a photosensitizer (PS), excitation light within the visible/near-infrared spectrum (400–700 nm), and molecular oxygen [17]. Its cytotoxic action is strictly oxygen-dependent, operating via Type I (free radicals/superoxide) or Type II (energy transfer yielding singlet oxygen, O21) pathways [17]. Cell death is mediated by biochemical oxidative damage to lipids, intracellular organelles, and tumor vascular structures.

Photothermal Therapy (PTT): A physical modality mediated by optical absorption by plasmonic or carbonaceous nanoparticles, organic dyes (e.g., indocyanine green), or conjugated polymers. Absorbed NIR photons undergo non-radiative vibrational relaxation, generating localized hyperthermia (Δ*T* ≥ 42–45 °C) [18]. The therapeutic effect is oxygen-independent, driven by thermal denaturation of cytosolic proteins, thermal disruption of cell membranes, and heat-shock-mediated collapse [18].

Bioengineered Nano-Photoimmunotherapy (Nano-PIT/Nanoparticle-Mediated PIT): Hybrid, multifunctional nanoplatforms (e.g., liposomes, metal–organic frameworks, cell-membrane-camouflaged vesicles) designed to combine optical transducers with immune checkpoint silencers, tumor microenvironment (TME) modulators, or exogenous damage-associated molecular pattern (DAMP) agonists [4,19]. While leveraging photodynamic or photothermal primary cytotoxicity, these nanoplatforms are engineered to orchestrate multi-step immune priming and bypass biological transport barriers (Table 1).

Throughout this review, the term “antibody-IR700 NIR-PIT” refers strictly to targeted antibody-IRDye700DX biophysical conjugates, whereas nanoparticle-based platforms employing photochemical or photothermal cytotoxic cascades are classified under “nanoparticle-mediated photo-immunotherapy” or their specific photophysical designation.

Given the convergence of optical physics, nanomedicine, cellular immunology, and surgical oncology within this field, this review is structured to accommodate readers of diverse clinical and scientific backgrounds. Readers focused primarily on subcellular biophysics and regulated cell-death subroutines are directed to Section 2 (molecular mechanisms of membrane lysis and DAMP trafficking) and Section 6 (the cGAS-STING innate axis). Materials scientists and pharmaceutical bioengineers may focus on Section 3, which details the architectural design of stimuli-responsive nanoplatforms, transition-metal complexes, and biomimetic vesicles. Clinical oncologists and translational immunologists may navigate directly to Section 4 (preclinical and clinical combinatorial regimens with immune checkpoint inhibitors, cytokines, and cellular therapies) and Section 7, which critically evaluates translational bottlenecks, clinical trial design, safety risks, and health-economic barriers.

## 2. Molecular Mechanisms of Photically Induced Immunogenicity

To critically evaluate photically induced immunogenicity, a fundamental distinction must be drawn between the mechanisms driving canonical antibody-IR700 NIR-PIT and those driving engineered photochemical/photothermal nanomedicines [8]. Canonical NIR-PIT (e.g., cetuximab-IR700) operates via an oxygen-independent, purely biophysical mechanism [9]. Photochemical cleavage of the hydrophilic axial ligands of IRDye700DX induces insolubility and rapid receptor aggregation on the plasma membrane, causing immediate physical shear stress, transmembrane water influx, and explosive osmotic cell burst within minutes [8]. In this classical modality, immunogenicity stems from the sudden, uninhibited release of intact, native tumor antigens and preformed cytosolic DAMPs without requiring intracellular ROS accumulation [11,20]. Conversely, organelle-localized oxidative stress (e.g., endoplasmic reticulum [ER] stress, mitochondrial outer membrane permeabilization), execution of regulated non-apoptotic death subroutines (pyroptosis, cuproptosis, ferroptosis), and sustained cGAS-STING innate cascades have been primarily decoded in nanoparticle-assisted PDT and PTT systems [21,22,23,24,25]. In these bioengineered platforms, intracellular photosensitizers or inorganic nanomaterials generate intense localized ROS fluxes (O21, OH∙) or hyperthermia upon illumination, intentionally engaging complex intracellular stress signaling pathways [21,25,26]. Clarifying this distinction is essential to avoid attributing intracellular photochemical cascades to canonical antibody-IR700 conjugates, which bypass intracellular biochemical processing entirely through direct mechanical lysis [9].

### 2.1. Subcellular Organelle Stress and the Induction of ICD

A rigorous dissection of immunogenic cell death (ICD) requires demarcating the distinct upstream cellular triggers operating in classical antibody-IR700 conjugates versus photochemical and photothermal nanomedicines. In organelle-targeted PDT and hybrid nanoplatforms, the primary cytotoxic stressor is the focal accumulation of ROS generated within specific subcellular compartments [13,27]. When photosensitizers localize selectively to the ER, light activation triggers the unfolded protein response (UPR) through protein kinase RNA-like ER kinase (PERK) activation Once activated, PERK phosphorylates eukaryotic translation initiation factor 2 alpha (eIF2α), arresting global protein translation while directing the pre-apoptotic trafficking of luminal chaperones [21]. Causal genetic validation has established that intact PERK-eIF2α signaling is indispensable for pre-lytic calreticulin (CRT) exposure in ER-targeted PDT, as cells harboring non-phosphorylatable eIF2α mutants or PERK knockouts fail to translocate CRT [21]. Similarly, photosensitizers engineered to accumulate in the mitochondrial matrix provoke targeted lipid peroxidation and catastrophic outer mitochondrial membrane permeabilization, releasing apoptogenic factors and triggering secondary lytic cascades. In sharp contrast, canonical antibody-IR700 NIR-PIT bypasses intracellular organelle stress cascades entirely [8,14]. Biophysical studies have causally demonstrated that upon 690 nm illumination, the photochemical cleavage of the hydrophilic axial ligands of IRDye700DX induces immediate insolubility, leading to the rapid aggregation of antibody-receptor complexes exclusively on the outer plasma membrane [8,14]. This physical aggregation exerts intense mechanical shear stress on the lipid bilayer, producing non-selective physical rupture within minutes [14]. Quantitative real-time imaging confirms that this biophysical burst triggers immediate, uncontrolled extracellular water and sodium influx, culminating in explosive osmotic swelling and necrotic oncosis [14]. Crucially, this cytolytic mechanism is functionally preserved under complete hypoxia and in the presence of potent intracellular ROS scavengers, causally confirming that canonical NIR-PIT does not depend on mitochondrial ROS generation, ER stress, or transcriptional eIF2α cascades [8,14]. The upstream trigger in canonical NIR-PIT is strictly biophysical and mechanical, whereas organelle stress cascades represent specialized photochemical mechanisms of organelle-homing nanomedicines.

### 2.2. Spatiotemporal Dynamics of Damage-Associated Molecular Patterns (DAMPs)

The immunogenicity of photically induced cell death is functionally dictated by the coordinated, spatiotemporally regulated exposure and release of DAMPs. These molecules, sequestered inside the healthy cell, are exposed on the outer leaflet of the plasma membrane or secreted into the extracellular space during organelle collapse. The key DAMPs involved in PIT-mediated ICD operate through distinct temporal phases:

CRT—The “Eat-Me” Signal: In healthy cells, CRT resides in the ER lumen, where it regulates calcium homeostasis and glycoprotein folding. In organelle-targeted PDT, CRT exposure is an active, pre-lytic event driven by ROS-mediated ER stress and PERK-eIF2α phosphorylation, translocating to the cell surface via a caspase-8- and BAP31-dependent exocytic pathway prior to plasma membrane permeabilization [21]. Conversely, in canonical antibody-IR700 NIR-PIT, CRT exposure does not require active vesicular trafficking or caspase-8 activation; rather, the instantaneous mechanical rupture of the plasma membrane physically exposes luminal and cytosolic CRT directly to the extracellular space, facilitating immediate recognition by CD91 receptors on dendritic cells [28,29].

Adenosine Triphosphate (ATP)—The “Find-Me” Signal: During photochemical PDT and PTT-induced stress, ATP is actively extruded into the extracellular environment during early cell death through caspase-dependent pannexin-1 (PANX1) channels or lysosomal exocytosis [30]. Subsequently, ATP binds to P2X7 receptors on mature DCs, activating the NLRP3 inflammasome and driving the secretion of pro-inflammatory cytokines such as IL-1β. In contrast, in antibody-IR700 NIR-PIT, ATP release is causally linked to explosive osmotic cell lysis: within 10–15 min of light exposure, the rapid loss of plasma membrane integrity causes a macroscopic, passive discharge of intracellular ATP pools into the tumor interstitial space, creating an immediate purinergic chemotactic gradient for P2Y2-expressing precursor dendritic cells [30].

High Mobility Group Box 1 (HMGB1)—The Adjuvant Signal: HMGB1 is a non-histone chromatin-binding protein structurally sequestered in the nucleus. During the late, lytic phase of photically induced cell death, compromised nuclear and plasma membrane integrity allows HMGB1 to passively diffuse into the extracellular space [30]. Extracellular HMGB1 binds primarily to Toll-like Receptor 4 (TLR4) on DCs, activating the MyD88-dependent signaling pathway. This interaction is essential for preventing the lysosomal degradation of phagocytosed tumor antigens within the DC, thereby ensuring optimal antigen processing and subsequent cross-presentation to T cells.

HSP (Hsp70/Hsp90)—Chaperone Facilitators: Photothermal and photodynamic stress stimulate the rapid upregulation and extracellular release of chaperone proteins Hsp70 and Hsp90 [31,32]. These chaperones bind directly to tumor-specific neoantigens, stabilizing them and forming immunogenic complexes. Upon release, they bind to CD91 and scavenger receptors on DCs, dramatically enhancing the efficiency of antigen internalization and MHC class I cross-presentation [31].

### 2.3. Non-Apoptotic Inflammatory Death Pathways: Pyroptosis, Cuproptosis, and Ferroptosis

While classical antibody-IR700 NIR-PIT induces rapid biophysical oncosis, recent research in bioengineered nano-photoimmunotherapy has focused on designing inorganic, organic, and biomimetic nanoplatforms specifically tailored to engage regulated, non-apoptotic lytic pathways. Unlike canonical NIR-PIT, which executes rapid osmotic membrane perforation independently of enzymatic death programs, these specialized nano-formulations bypass apoptotic resistance by deliberately executing three distinct inflammatory cascades:

Pyroptosis: Characterized by cell swelling, large membrane blebbing, and rapid lysis, pyroptosis is executed downstream of targeted photochemical triggers. Nanoparticles engineered to accumulate in specific organelles, such as macrophage-membrane-camouflaged AIE nanoplates targeting the Golgi apparatus (EM@TCN), generate intense focal ROS that activate caspase-3/8 or caspase-1, leading to Gasdermin D (GSDMD) or (Gasdermin E) GSDME cleavage and pore formation with the release of mature IL-1β and IL-18 [22,23].

Cuproptosis: Cuproptosis represents a copper-dependent non-apoptotic modality mediated by protein lipoylation [23]. This pathway has been demonstrated using copper-doped hollow nanoplatforms and sulfidation-activated carbon nitride nanocomposites under NIR light, rather than standard antibody conjugates [23,24]. Photothermal and photodynamic activation triggers the intracellular release and accumulation of cupric ions (${\mathrm{Cu}}^{2+}$) that selectively target lipoylated components of the tricarboxylic acid (TCA) cycle, specifically dihydrolipoamide S-acetyltransferase (DLAT). This copper-dependent proteotoxic stress drives the toxic oligomerization of lipoylated mitochondrial enzymes and the destabilization of iron-sulfur (Fe-S) cluster proteins, culminating in acute mitochondrial proteotoxic collapse and immunogenic lytic cell demise [23,24].

Ferroptosis: Ferroptosis requires iron-dependent lipid peroxidation [25]. Experimental evidence for light-triggered ferroptosis is derived from mitochondria-targeted transition-metal complexes (e.g., lipophilic cationic iridium(III) complexes) and dual-enzyme nanozymes, where photostimulation triggers ferritinophagy, an influx of free intracellular ${\mathrm{Fe}}^{2+}$, and Fenton-driven lipid membrane oxidation [25,33].

### 2.4. Antigen Presentation, Dendritic Cell Priming, and CD8+/CD4+ T-Cell Activation

The ultimate translational goal of photically induced ICD is to translate a localized physical intervention into a durable, systemic antitumor immune response. This transition is mediated by the cancer-immunity cycle, which is initiated immediately following the lytic destruction of the primary tumor [13]. The localized burst of ATP acts as a homing beacon, recruiting immature iDCs directly into the newly inflamed tumor site [30]. Upon arrival, these DCs utilize their pattern recognition receptors (PRRs), such as TLR4 and CD91, to bind the exposed CRT and extracellular HMGB1-antigen complexes. This signaling cascade stimulates highly efficient phagocytosis, prompting the DCs to internalize tumor-associated antigens (TAAs) and somatic neoantigens [28]. Concurrently, the pro-inflammatory cytokine milieu (IL-1β, TNF-α, IFN-β) drives the maturation of iDCs into antigen-presenting cells (mDCs), characterized by the upregulation of co-stimulatory molecules (CD80, CD86, and CD40) and chemokine receptors (such as CCR7) [34]. These mature DCs migrate via the lymphatic system to the TDLNs. Within the lymph nodes, they execute antigen cross-presentation: TAAs are loaded onto MHC Class I molecules for presentation to naive CD8+ T cells, and onto MHC Class II molecules for presentation to naive CD4+ T cells [34]. This precise priming and clonal expansion generates antigen-specific CTLs and T helper 1 (Th1) effector cells. Once activated, these effector T cells enter the systemic circulation and traffic back to the primary tumor as well as to distant, non-irradiated metastatic lesions. This process mediates the systemic abscopal effect in preclinical tumor models, establishing a robust immunological memory that protects the host against subsequent tumor rechallenge [13,34]. Additionally, this inflammatory cascade remodels the local immune infiltrate by functionally reprogramming immunosuppressive tumor-associated macrophages (TAMs) from a pro-tumorigenic, M2-like phenotype toward an immunostimulatory, tumoricidal M1-like activation state across the macrophage polarization spectrum [29], further breaking down local immunological tolerance.

## 3. Advanced Bioengineered Nano-PIT Platforms and Smart Delivery

### 3.1. Mitochondria-Targeted Transition Metal Complexes for Hypoxia-Independent Therapy

The clinical translation of conventional photodynamic therapies is systematically hindered by severe hypoxic gradients within desmoplastic solid tumors. To address this biological constraint, bioengineered nanomedicines have leveraged transition metal complexes with unique photophysical properties, particularly those based on iridium(III) and ruthenium(II) scaffolds, to bypass the requirement for ground-state molecular oxygen [35]. Cationic iridium(III) complexes exhibit exceptionally high phosphorescence quantum yields, large Stokes shifts, and prolonged triplet-state lifetimes, which allow them to undergo robust photoinduced electron transfer (Type-I photochemical reactions) directly with intracellular substrates, generating toxic hydroxyl (•OH) and superoxide (O_2_^•−^) radicals even in hypoxic conditions [35]. A crucial strategy in modern transition metal design involves sub-organelle targeting, specifically directing these complexes to the mitochondria. Due to the high negative mitochondrial membrane potential, lipophilic cationic complexes accumulate selectively within the mitochondrial matrix. When illuminated, these agents disrupt the mitochondrial respiratory chain and induce localized lipid peroxidation. Specifically, an iridium(III)-based PS targeting the mitochondrial respiratory chain has been shown to induce ferritinophagy, the autophagic degradation of the iron-storage protein ferritin [25]. This ferritinolysis triggers a massive influx of free intracellular ferrous iron (Fe^2+^), which drives the Fenton reaction to generate hydroxyl radicals, leading to highly immunogenic lipid-peroxidation-mediated cell death [25]. This mechanism bypasses the traditional apoptotic pathways and promotes robust ICD. To improve biodistribution and reduce systemic toxicities, these iridium complexes are integrated into complex nanocarriers and biomimetic vesicles. For instance, the encapsulation of the cationic iridium(III) complex (IrC8) into giant plasma membrane vesicles (GPMVs) derived from cancer cells yields a biomimetic hybrid platform (G-IrC8) that retains homotypic targeting capabilities [36]. These bioengineered GPMVs facilitate precise, enhanced accumulation of the metal complex within the target tumor parenchyma in vivo. Upon NIR activation, the G-IrC8 platform induces massive mitochondrial ROS production, triggering rapid membrane lysis, ICD, and the consequent maturation of local DCs [36]. In addition to transition metal complexes, structural self-assembly of the PSs themselves has emerged as a powerful ‘carrier-free’ delivery concept. The development of a mitochondria-targeting, full-active pharmaceutical ingredient (full-API) nanosensitizer represents a highly efficient formulation [37]. Composed entirely of active therapeutic agents without inert carrier matrices, these full-API self-assembled nanoagents avoid vehicle-associated systemic toxicity. By targeting mitochondria directly, these self-assembled nanosensitizers generate exceptionally high local concentration of ROS, leading to robust mitochondrial dysfunction, cytocidal action, and highly immunogenic cancer cell destruction [37].

### 3.2. Biomimetic Platforms and Immune-Derived Exosomes for Targeted PIT

Natural extracellular vesicles (EVs) and exosomes derived from specific cellular subsets present an elegant biomimetic approach for targeted photoimmunotherapy, combining excellent biocompatibility, low immunogenicity, and long systemic circulation with natural tumor-homing properties. A primary breakthrough in this domain is the engineering of photosensitive hybrid exosomes derived from γδ-T cells (gamma-delta T cells) [38]. These hybrid exosomes inherit the innate homing capability of γδ-T cells toward cancer cells via chemokine receptors (such as CCR5) and tumor-homing molecules, and retain endogenous cytotoxic molecules (granzyme B, perforin, and granulysin). Upon NIR irradiation, the co-loaded PSs generate localized ROS that act in synergy with these cytotoxic proteins, inducing a highly immunogenic necrotic cascade that triggers robust dendritic cell maturation and expands tumor-specific antigen-reactive T cell clones, establishing durable immunological memory [38]. Similarly, plant-derived extracellular vesicle platforms have emerged as versatile carrier systems [39]. In a representative primary study, bioengineered plant-derived exosome-like nanovesicles functionalized with metal–organic frameworks (such as ZIF-8), photosensitizers (Ce6), and small interfering RNA targeting PD-L1 were developed to combine biocompatibility with dual optical and gene-silencing therapies [40]. In this platform, the plant vesicular shell facilitates tumor-targeted biodistribution, while co-delivered siRNA silences checkpoint evasion and the light-activated photosensitizer triggers photodynamic ICD, promoting robust dendritic cell maturation within tumor-draining lymph nodes [40]. Concurrently, biomimetic platforms utilizing cancer-cell-derived membranes, such as melanoma MHC-I-camouflaged nanoparticles encapsulating copper and ferrihydrite, provide targeted induction of synchronized ferroptotic and cuproptotic death cascades, amplifying systemic tumor-specific immunity [19]. To remodel the dense, desmoplastic stromal-immune microenvironment, EVs have been engineered to target specific stromal receptors. Vescicles targeted to the midkine (MDK) receptor, such as the Ce6@D4-EV platform, selectively home to MDK-overexpressing cancer cells and active CAFs [41]. Upon light activation, the localized ROS generation induces selective cytotoxicity that degrades the dense stromal extracellular matrix (ECM, collagen and fibronectin), while the released tumor-specific dsDNA fragments engage the host cGAS-STING pathway, leading to robust IFN-β production and profound remodeling of the immunosuppressive TME [41]. To orchestrate the active infiltration of immune cells, exosomes have been functionalized with immunostimulatory chemokines. For example, NIR-responsive exosomes loaded with the chemokine CXCL9 and the PS cypate (cypate@EXO-CXCL9) establish a strong localized chemokine gradient in the tumor bed post-irradiation [42]. This CXCL9 gradient drives the active recruitment of CXCR3+ CTLs and NK cells into the tumor parenchyma. Furthermore, the combined phototoxicity and chemokine release promote the functional re-education of TAMs from an immunosuppressive M2-like phenotype toward an active, tumoricidal M1-like polarization profile [42]. In the context of oral squamous cell carcinoma (OSCC), homotypic tumor-derived exosomes (TEX) have been harnessed to bypass biological barriers. A dual-loaded homotypic TEX platform (ID-TEX) incorporating the near-infrared heptamethine dye IR808 and pH-responsive, hydrazone-linked doxorubicin (DOX) provides coordinated sequential chemo-phototherapy [43]. The homotypic membranes allow ID-TEX to target parent OSCC cells through self-recognition adhesion molecules. Upon cellular internalization, the acidic lysosomal environment triggers the cleavage of the hydrazone bonds, releasing DOX to induce nuclear DNA damage, which enhances IR808-mediated phototoxicity under NIR light, resulting in increased ICD [43]. Further, advanced hybrid biomimetic nanoplatforms, such as macrophage-derived EVs fused with cell membrane hybrids (EM@TCN), have been engineered to target specific intracellular organelles. By coating an aggregation-induced emission (AIE) PS with membrane hybrids, the EM@TCN platform selectively targets the Golgi apparatus [22]. Under light stimulation, the Golgi-localized ROS generation triggers immediate organelle swelling, disrupting cellular transport and initiating a coordinated cascade of GSDMD-dependent pyroptosis and classical ICD, culminating in a robust systemic antitumor immune response and mature dendritic cell presentation [22].

### 3.3. Smart-Delivery Systems: Transdermal Microneedles and Responsive Assemblies

For superficial and accessible malignancies such as melanoma, transdermal microneedle patches (MN) represent a groundbreaking minimally invasive delivery modality. Traditional transdermal delivery is hampered by the stratum corneum barrier and severe hypoxic conditions in the subcutaneous tumor bed. To address this, a smart DNA-nanoflowers-based microneedle patch (Z/H@DFMNs) has been engineered for synergistic transdermal therapy [44]. This system incorporates an artificial DNA-zyme (G-quadruplex/hemin) that catalytically cleaves endogenous tumor-derived hydrogen peroxide (H_2_O_2_) to generate molecular oxygen (O_2_) in situ, actively reversing the hypoxic state of the tumor. This newly generated oxygen fuels the co-loaded zinc phthalocyanine (ZnPc) PS to produce high concentrations of singlet oxygen, while the simultaneous transdermal release of PD-L1 antisense oligonucleotides (ASO) blocks immune checkpoint evasion, achieving potent synergistic efficacy [44]. Similarly, a redox-responsive microneedle patch has been engineered to deliver a combination of epigenetic and photodynamic agents [45]. Composed of glutathione (GSH)-sensitive hyaluronic acid, the microneedles dissolve rapidly upon penetrating the epidermis, releasing the hypomethylating drug decitabine and redox-responsive HPPH-loaded nanoparticles (HPPH-ss-NPs). The GSH-induced degradation of the nanoparticles triggers the rapid intracellular release of the HPPH PS. Under light irradiation, this platform triggers immediate GSDME-mediated pyroptosis, which is further sensitized by the epigenetic action of decitabine, transforming the immunosuppressive melanoma microenvironment into an immunologically active site [45]. To enhance penetration depths of therapeutic agents in bulky subcutaneous tumors, active self-propelled systems have been integrated into microneedle technologies [46]. A novel self-propelled gas nanomotor-integrated microneedle system utilizes magnesium (Mg) micromotors to actively drive transport within the tumor bed [46]. Upon transdermal penetration, the Mg nanomotors react with the interstitial fluid to generate hydrogen ($H_{2}$) gas bubbles, which act as a powerful mechanical propellant that actively drives the co-loaded PSs and immunoadjuvants deep into the dense, desmoplastic tumor parenchyma, ensuring uniform light activation and overcoming the physical limitations of passive diffusion. In addition to microneedles, responsive self-assembling smart polymers are highly effective for deep-tissue photoimmunotherapy. A sequentially activated smart DNA nanosphere system utilizes a pH-responsive polymeric core for the coordinated delivery of PSs and PD-L1 siRNA [47]. The DNA nanospheres remain stable at physiological pH, but undergo rapid protonation and swelling within the acidic lysosomal and TME. This structural dissociation triggers the sequential release of the PS and PD-L1 siRNA, ensuring that PDT and ICB are activated with high spatial and temporal precision, drastically reducing off-target systemic toxicities [47]. Finally, the concept of peptide supramolecular assembly has been applied to induce in situ self-aggregation of therapeutic agents. A peptide supramolecular system has been engineered to respond to specific enzymatic or physical triggers within the TME, initiating self-aggregation into dense nanofibers [48]. This localized phase transition restricts the diffusion of co-assembled AIE luminogens, preventing their clearance from the tumor bed and significantly enhancing their photostability and local ROS generation, thereby maximizing the therapeutic index of optical immunotherapies [48] (Table 2).

## 4. Combinatorial Integration with Systemic Immunotherapies: Additive Benefit, Functional Cooperativity, and Synergy

While local phototherapies are highly effective at ablating primary lesions, their utility as monotherapies is strictly bounded by their localized nature. The modern oncological paradigm recognizes that to prevent metastatic relapse and treat disseminated disease, local intervention must be seamlessly integrated with systemic immunotherapies. This chapter examines the mechanical, cellular, and therapeutic synergy resulting from the combination of localized phototherapy (photoimmunotherapy [PIT] and photothermal therapy [PTT]) with systemic immunological modalities. We analyze the integration of light-induced immunogenic ablation with ICB, cytokine-adjuvanted local in situ vaccination, and the priming of adoptive cellular therapies (ACT). From a translational and pharmacological perspective, a rigorous distinction must be maintained between an enhanced combinatorial benefit (where a combination arm achieves statistically superior tumor reduction over single-agent controls), an additive effect (independent action conforming to Bliss independence or Loewe additivity models), and true pharmacological synergy (where the combined effect exceeds the mathematical sum of individual monotherapy contributions, as verified by formal Combination Index algorithms, CI<1) [49,50]. In immuno-oncology, superior in vivo outcomes frequently reflect mechanistic cooperativity, wherein local photic debulking physically reduces tumor burden and releases native neoantigens, thereby lowering the threshold required for systemic checkpoints or transferred T cells to overcome exhaustion, rather than supra-additive pharmacological synergy in the classical sense [49]. Throughout this chapter, the term “synergy” is restricted to regimens supported by formal interaction modeling or demonstrated non-redundant mechanistic cross-talk, whereas combinations demonstrating improved therapeutic outcomes without formal mathematical interaction metrics are designated as displaying enhanced combinatorial efficacy or additive antitumor action [50].

### 4.1. Translational Framework: Clinical Status vs. Preclinical Proof-of-Concept

When evaluating combinatorial photo-immunotherapy, it is essential to rigorously distinguish established clinical milestones from emerging preclinical strategies. To date, regulatory approval of NIR-PIT is limited to cetuximab sarotalocan monotherapy (ASP-1929, targeting EGFR), approved in Japan for patients with locally advanced or recurrent HNSCC [6,7]. Moving from monotherapy to combination regimens, early-phase clinical trials are currently investigating the combination of cetuximab sarotalocan with systemic immune checkpoint blockade (such as anti-Programmed Cell Death Protein 1 [PD-1] antibodies) [51], alongside early-phase evaluations of novel light-activated agents such as virus-like drug conjugates (e.g., Bel-sar in bladder cancer). Conversely, the vast majority of multi-modal combinatorial strategies discussed below, including dual checkpoint co-blockade (CTLA-4, LAG3), synthetic STING agonist delivery, intratumoral cytokine vaccination (IL-15, IL-2), and adoptive cell therapy priming, currently represent preclinical proof-of-concept studies conducted in syngeneic murine and immunocompetent animal models [15,52,53,54,55,56,57,58]. Clarifying this translational boundary is critical to avoid premature clinical extrapolation while defining the prospective roadmap for multimodal oncological translation.

### 4.2. Combinatorial ICB Synergy and the Systemic Abscopal Effect

The primary objective of combining local phototherapies with systemic ICB is to convert immunologically ‘cold’ tumors into ‘hot’, T-cell-inflated niches, thereby triggering the systemic regression of non-irradiated distant metastases, a phenomenon known as the abscopal effect. Translational studies conducted by Rakuten Medical have confirmed that NIR-PIT induces rapid and super-selective tumor necrosis in vivo, and that the subsequent systemic addition of anti-PD-1 antibodies prevents long-term tumor recurrence and establishes a durable immune memory [51]. NIR-PIT-induced cell destruction serves as a potent therapeutic vaccine in situ, while PD-1/PD-L1 axis blockade prevents clonal exhaustion of infiltrating CD8+ CTLs, amplifying the magnitude of the adaptive antitumor response [51]. However, the efficacy of PD-L1 blockade is closely related to baseline levels of checkpoint expression in the TME, which is often deficient in cold tumors. To overcome this limitation, a frontier study showed that pre-treatment of immunocompetent tumors with pegylated interferon-gamma (IFN-γ) markedly increases PD-L1 expression on both tumor cells and infiltrating immune cells. This preconditioning dramatically sensitizes tumor lesions to subsequent PD-L1-targeted NIR-PIT (anti-PD-L1-PIT), leading to increased CD8+ lymphocyte infiltration and high rates of complete tumor regression in immunocompetent murine models [52]. In addition, the adoption of repeated cycles of porphyrin-lipoprotein-mediated photodynamic therapy (R-PDT) has shown the induction of robust innate immune activation (characterized by a more than 40-fold increase in IL-6) and adaptive immune activation, which, when combined with systemic anti-PD-1, significantly broadens the systemic abscopal effect compared to either monotreatment alone [59].

In addition to the classic PD-1/PD-L1 axis, recent research has focused on the co-inhibition of alternative and synergistic immunosuppressive immune checkpoints, such as CTLA-4 and LAG3. Simultaneous administration of cancer cell-targeted NIR-PIT (EGFR targeting) and immune cell-targeted NIR-PIT (using an anti-CTLA-4-IR700 conjugate for focal depletion of intratumoral CD4+CD25+ Foxp3+ Treg demonstrated marked combinatorial antitumor efficacy in preclinical models, achieving cooperative therapeutic activity by simultaneously inducing immediate primary tumor lysis and eliminating the local immunosuppressive brake imposed by Tregs [15]. In parallel, the selection of CD29 (beta-1 integrin) as an antigenic target for NIR-PIT showed that this target preserves non-antigen-expressing infiltrating immune cells, and its dual combination with systemic anti-CTLA-4 antibodies produces robust remission and immune protection against tumor rechallenge in highly pigmented melanoma models [60]. In addition, the integration of phototherapies that induce highly inflammatory cell death mechanisms such as PANoptosis with blockade of the emerging LAG3 checkpoint has been shown to favorably reprogram the immune niche, leading to systemic suppressor myeloid cell suppression (MDSC) and robust increase in dendritic type 2 cells (cDC2) and CD8+ CTLs [53] (Table 3 and Table 4).

### 4.3. Biological Heterogeneity and Preclinical Limitations of the Abscopal Effect

While the induction of systemic abscopal regression—defined as the immunologically mediated shrinkage of non-irradiated distant lesions following local ablation—remains a cornerstone ambition of combinatorial phototherapy, its reliability must be evaluated with scientific caution [61]. In clinical oncology, bona fide abscopal responses following focal interventions remain exceedingly rare and unpredictable [61]. Most evidence supporting robust abscopal remissions in photo-immunotherapy derives from bilateral or metastatic syngeneic murine models (e.g., bilateral MC38 colon or bilateral TC-1 tumors) [15,51]. These experimental models often possess high baseline antigenicity, homogeneous target receptor overexpression, and predictable vascular beds that poorly reflect the complex spatial heterogeneity of human advanced metastases [12]. The capacity of phototherapy to successfully convert an immunologically “cold” lesion into an inflamed “hot” phenotype is strictly tumor-type dependent [12]. In malignancies characterized by severe intrinsic immune desert phenotypes, such as low tumor mutational burden (TMB), genetic loss of $\beta_{2}$-microglobulin ($\beta_{2}M$), structural defects in the transporter associated with antigen processing (TAP1/2), or major histocompatibility complex class I (MHC-I) transcriptional downregulation, the photic extrusion of intracellular antigens cannot restore CD8+ T-cell recognition [12,62]. Furthermore, in highly desmoplastic tumors such as pancreatic ductal adenocarcinoma, extensive dense stroma and hyperactivated systemic myeloid-derived suppressor networks can rapidly extinguish photically primed T cells before systemic trafficking to distant metastases can occur [12,63]. Therefore, light-induced tumor debulking must be recognized not as an absolute immunological switch, but as an in situ priming event whose systemic therapeutic outcome is fundamentally conditioned by the underlying immuno-genomic architecture of the host and tumor [12,61].

### 4.4. Cytokine-Adjuvanted In Situ Vaccination and Immunostimulatory Nanomedicines

To maximize the efficacy of light-induced ICD, investigators have engineered multimodal strategies that combine physical tumor ablation with the localized delivery of immunostimulatory cytokines or nanomedicines, functioning as highly potent in situ cancer vaccines. A prominent avenue involves the administration of interleukins directly into the photically disrupted tumor bed. The combination of CD25-targeted NIR-PIT (to locally deplete Treg cells) with intratumoral administration of Interleukin-15 (IL-15) has been shown to activate innate and adaptive host immunity, substantially attenuating primary resistance mechanisms to systemic anti-PD-1 therapy in preclinical settings [54]. Intratumoral IL-15 serves as a survival and proliferation factor for cytotoxic CD8+ T lymphocytes and NK cells, prolonging the functional activity of antigen-specific CD8+ effector T cells previously induced by tumor lysis [54]. Similarly, the addition of IL-15 to tumor NIR-PIT massively increases the density of NK and CD8+ T cells at treated tumor sites, significantly inhibiting the development of spontaneous lung metastases [55]. This combinatorial therapeutic benefit is similarly observed using gold nanorod-mediated PTT combined with immune-stimulating cytokines such as intratumoral IL-2, which favorably modulates the balance between cytotoxic effector T cells and Treg cells at untreated metastatic sites, confirming the systemic extension of tumor immunity [55]. The integration of immunological adjuvants within intelligent nanotechnology platforms has made it possible to temporally coordinate antigenic presentation and activation of Toll-Like Receptors (TLRs). The development of a flagellin-adjuvanted (FlaB) heat-resistant in situ vaccine encapsulated in targeted liposomes loaded with indocyanine green (ICG) demonstrated that, following local PTT, controlled release of FlaB potently stimulates TLR5s on DCs. This activation, when cooperatively paired with systemic anti-PD-1 treatment, induces a systemic tumor regression of 90% and protects subjects from orthotopic tumor rechallenge [56]. Similarly, the association of PDT with immune-stimulating polymeric nanoparticles loaded with CpG agonists (TLR9 ligand) and tumor peptides dramatically accelerates the anti-cancer immune cycle, stimulating antigenic cross-presentation and resulting in a prolonged survival rate in vivo [64]. Finally, the advent of phototherapeutic agents operating in the second biological window of the near-infrared (NIR-II, 1000–1350 nm) has made it possible to overcome the limits of tissue penetration. The development of the Ag@CuS-TPP@HA nanocomposite has shown that photothermal in NIR-II coupled with amplified ROS generation induces robust ICDs levels, which in combination with systemic PD-L1 blockade significantly suppressed pulmonary metastatic burden in triple-negative breast models [65].

### 4.5. Priming and Potentiating Adoptive Cellular Therapies (ACT)

Adoptive Cell Therapy (ACT), including CAR T-cell, Natural Killer (NK) cell, cell infusions, represents a highly promising systemic immunotherapeutic paradigm, yet its clinical efficacy in solid tumors is severely limited by poor cell homing, the physical barrier of the dense ECM, and an immunosuppressive TME. Recent studies have demonstrated that local phototherapy can serve as a powerful ‘primer’ to facilitate and enhance adoptive cellular therapies. In a pioneering approach, tumor-specific T cells were expanded and engineered ex vivo using tumor cells undergoing Prussian blue nanoparticle-induced photothermia as an antigenic source. This protocol highlighted that PTT preserves the structural integrity of native antigens released during ICD, providing DCs with ideal stimuli to induce an ex vivo expansion of specific T lymphocytes endowed with a highly cytotoxic phenotype and long-term memory for adoptive immunotherapy [57]. Moreover, the application of PIT to target specific antigens such as ganglioside GD2 has demonstrated not only the direct ablation of neuroblastoma tumor cells, but also a drastic stromal vascular remodeling that facilitates the infiltration and survival of adoptive NK and NKT cells previously blocked by the physical barriers of the ECM, paving the way for promising translational combinatorial therapies [58].

## 5. Overcoming Immunosuppression in the Tumor Microenvironment

### 5.1. Selective Photic Depletion of Immunosuppressive Cell Populations

Representing the archetype of direct immune-targeted photoimmunotherapy, localized photic depletion selectively ablated regulatory immune cells rather than malignant tumor cells [14,16,54]. The therapeutic success of localized phototherapy is frequently limited by the presence of highly immunosuppressive cell populations that reside within the TME. Among these, regulatory T cells (Tregs) expressing the CD25 receptor and polymorphonuclear myeloid-derived suppressor cells (PMN-MDSCs) represent key cellular shields that tumors utilize to shut down cytotoxic T-cell responses. Recent breakthroughs in NIR-PIT have enabled the development of highly selective, cell-type-specific depletion strategies to dismantle these barriers directly within the treated tumor bed without triggering systemic autoimmunity [15,16]. Traditional systemic depletion of Tregs often results in severe, life-threatening autoimmune side effects due to the systemic loss of self-tolerance. To bypass this limitation, localized CD25-targeted NIR-PIT has been engineered to eliminate CD4+CD25+ Tregs selectively within the illuminated tumor site [16]. Upon binding an anti-CD25-IR700 conjugate and applying 690 nm NIR light, intratumoral Tregs undergo rapid biophysical membrane rupture and necrotic lysis. This highly localized depletion selectively removes the Treg-mediated suppression of effector T cells (Teffs) and NK cells exclusively in the local TME, facilitating their reactivation and proliferation [16]. Crucially, because normal circulating Tregs and those in lymphatic organs are spared, this approach controls local immune suppression without inducing systemic autoimmune toxicities. In addition to Tregs, particularly polymorphonuclear (PMN-MDSCs), represent another major barrier, secreting high levels of immunosuppressive factors such as arginase-1, ROS, and nitric oxide. To address this cellular shield, Kato and colleagues successfully developed a NIR-PIT strategy targeting the Ly6G receptor using an anti-Ly6G-IR700 conjugate [15]. The localized ablation of PMN-MDSCs via anti-Ly6G NIR-PIT led to an immediate reduction in the local suppressive secretome, which subsequently restored the cytotoxic activity of CD8+ T cells and NK cells [15]. This cellular depletion approach represents a powerful method to reprogram the local immune infiltration, tilting the balance of the TME from a deeply suppressed state to a highly active, tumoricidal state.

### 5.2. Deconstructing Tolerogenic Metabolic and Enzymatic Pathways

Beyond cellular barriers, the TME is reinforced by complex metabolic and enzymatic circuits that actively metabolize nutrients, deplete key amino acids, and generate immunosuppressive metabolites. Prominent among these pathways are the indoleamine 2,3-dioxygenase 1 (IDO1) pathway, the cyclooxygenase-2/prostaglandin E2 (COX-2/PGE2) axis, and the ectonucleotidase CD73/adenosine cascade. Novel bioengineered phototherapeutic platforms are now being designed to deliver chemical inhibitors or genetic knockout tools in a spatiotemporally controlled manner, effectively silencing these metabolic escape routes [37,66,67,68]. The COX-2/PGE2 metabolic axis is frequently upregulated in solid tumors, particularly following the stress induced by PDT and PTT. The sudden generation of sublethal ROS or heat stimulates the expression of COX-2, leading to the rapid synthesis and secretion of PGE2, which suppresses dendritic cell maturation and impairs CTL infiltration. To counteract this compensatory immunosuppression, Xu and co-workers developed a synergistic nanomedicine that co-delivers a PS and the COX-2 inhibitor diclofenac [67]. By shutting down the PGE2-mediated signaling pathway, this platform effectively remodeled the PDT-associated immunosuppressive loop and enhanced the therapeutic efficacy of systemic anti-PD-L1 checkpoint blockade [67]. Similarly, Wang and colleagues engineered a dual-functional single-component prodrug (DHU-CBA2) that is activated specifically by tumor-endogenous hypochlorous acid (HOCl) to release the COX-2 inhibitor indomethacin and methylene blue [69]. This autoboosting strategy provided simultaneous COX-2 inhibition and IDO1 pathway inactivation, creating a metabolic blockade that reinforced optical tumor ablation. Another critical escape mechanism is the IDO1 pathway, which metabolizes the essential amino acid L-tryptophan into the immunosuppressive metabolite kynurenine, promoting regulatory T-cell differentiation and inducing effector T-cell anergy. Lin and colleagues designed multifunctional core–shell nanocapsules conjugated with anti-PD-L1 antibodies and loaded with both a PS and the IDO1 inhibitor NLG919 [66]. Upon near-infrared laser irradiation, this nanoplatform achieved precise photothermal ablation while simultaneously releasing NLG919 to block kynurenine synthesis, effectively rescuing anergic T cells and promoting their proliferation in triple-negative breast cancer (TNBC) models [66]. To address the extracellular accumulation of adenosine, which is generated from extracellular ATP by the ectonucleotidases CD39 and CD73 and acts as a powerful immunosuppressive signal through adenosine A2A receptors on T cells, researchers have developed CD73-targeted delivery platforms. Li and co-workers designed a biomimetic nanodrug (AptEM@CBA) that actively targets CD73 and co-delivers the CD73 inhibitor AMPCP alongside a photothermal agent [70]. This targeted CD73 blockade prevented the enzymatic conversion of immunogenic extracellular ATP (released during photically induced ICD) into immunosuppressive adenosine, maintaining a highly immunostimulatory extracellular environment [70]. Taking metabolic reprogramming a step further, Ning and colleagues developed an advanced platform integrating AIE PSs with CRISPR/Cas9 gene-editing machinery to achieve permanent genetic knockout of CD73 in glioblastoma cells [68]. By genetically silencing CD73 expression, this nanoplatform abolished adenosine generation permanently, enhancing the durable host antitumor immune response and reinforcing the therapeutic efficacy of photoimmunotherapy [68].

### 5.3. Targeting and Dismantling the Desmoplastic Tumor Stroma

The physical ECM of desmoplastic tumors, characterized by dense collagen fibers, hyaluronic acid deposition, and high IFP, acts as a dense barrier that restricts the infiltration of cytotoxic T cells and the penetration of therapeutic nanoparticles. CAFs are the primary architects of this ECM, actively depositing structural proteins and secreting pro-survival cytokines. Deconstructing this stromal barrier represents a key step toward improving therapeutic perfusion and facilitating systemic immune cell infiltration [63,71,72]. To eliminate CAFs selectively, Nishimura and co-workers developed a NIR targeting the Fibroblast Activation Protein (FAP), a cell-surface marker highly overexpressed on active CAFs [71]. The administration of an anti-FAP-IR700 conjugate followed by 690 nm NIR light irradiation induced highly specific, rapid lytic death of CAFs in vivo. This selective CAF ablation led to a drastic reduction in the local synthesis of collagen type I and hyaluronic acid, effectively decompressing the tumor stroma and lowering the high IFP [71]. As a direct consequence, the intratumoral penetration and therapeutic efficacy of subsequent systemically administered chemotherapeutic macromolecules (such as paclitaxel-bound albumin nanoparticles, Abraxane) were significantly enhanced. Similarly, Matsuoka and colleagues investigated the clinical translation of this approach by targeting FAP and podoplanin (PDPN) on CAFs in pancreatic ductal adenocarcinoma (PDAC) models, demonstrating that stromal deconstruction successfully restored tumor perfusion and improved therapeutic delivery [63]. To complement the photic depletion of CAFs, chemical approaches to remodel the ECM have been successfully integrated with phototherapy. Zhang and co-workers evaluated a combination therapy using Losartan, an angiotensin II receptor blocker clinically known to inhibit collagen synthesis, alongside platinum-based nanoparticles and PTT [72]. The administration of Losartan reduced the collagen density and normalized the tumor vasculature, which significantly improved the diffusion and intratumoral distribution of platinum nanoparticles. Under subsequent laser irradiation, this uniform nanoparticle distribution ensured homogeneous photothermal ablation and robust ICD induction in neuroblastoma models [72]. In another approach, Wu and co-workers engineered T-cell membrane-coated polydopamine nanoparticles loaded with triptolide (ETM@PT) to reverse tumor thermo-resistance [73]. This biomimetic system successfully targeted active CAFs, down-regulated the expression of matrix metalloproteinase-9 (MMP9) and prevented the heat-induced activation of protective heat-shock response pathways in the stroma, rendering the tumor highly sensitive to thermal ablation and subsequent immune attack [73].

### 5.4. Remodeling Immunosuppressive Cell Crosstalk and Hypoxia Cascades

To maximize the efficacy of photodynamic and photothermal therapies, advanced multifunctional nanomedicines are now designed to target the crosstalk between cancer cells and immunosuppressive cells, while simultaneously alleviating the hypoxia-driven signaling cascades that govern TME resistance [33,74,75]. The integration of PD-L1 degradation with PTT represents an elegant method to disrupt the PD-1/PD-L1 inhibitory axis on a cellular level. Tan and co-workers developed biomimetic platelet-membrane-coated thermosensitive liposomes designed to target PD-L1 and release PSs in response to heat generated by ICG [74]. This localized hyperthermia not only ablated the primary tumor cells but also stimulated the photothermal degradation of membrane-bound PD-L1, preventing immune escape and restoring CTL-mediated cytotoxicity. To target broader immunosuppressive networks, Cheng and colleagues engineered a novel gold nanostructure (AuNH-2-Ab) that releases anti-PD-L1 antibodies locally in response to the acidic pH and hyperthermia of the TME [75]. This pH/thermal dual-responsive release dismantled the crosstalk between tumor cells and TAMs, the phenotypic shift in macrophages toward a pro-tumoral M2-like state and enhancing NK-cell-mediated surveillance [75]. Finally, to address the hypoxia-induced stabilization of HIF-1α and the associated upregulation of heat-shock protein 70 (HSP70), which collectively mediate tumor resistance to ferroptotic and thermal therapies, Chang and co-workers developed a dual-enzyme-like nanozyme adjuvant co-encapsulated with melatonin (MLT) [33]. The local, enzymatic generation of oxygen relieved tumor hypoxia, while melatonin actively suppressed the expression of both HIF-1α and HSP70. This multi-pronged adjuvant therapy reversed the protective thermo-resistance of the TME and enhanced the therapeutic efficacy of photothermal-ferroptosis combined therapy, eliciting a robust and durable systemic antitumor immune response [33]. Table 5 provides a comprehensive summary of the cellular, metabolic, and stromal barriers addressed in this chapter, highlighting the specific platforms, mechanisms, and immunological outcomes.

## 6. Activation of the cGAS-STING Pathway and Innate Immunity

### 6.1. Photically Induced Mitochondrial DNA (mtDNA) Release as an Endogenous cGAS Stimulator

The cyclic GMP-AMP synthase-stimulator of interferon genes (cGAS-STING) pathway serves as a fundamental biological bridge linking localized ICD with systemic adaptive antitumor immunity. Historically, the cGAS-STING cascade evolved as a cell-intrinsic defense mechanism designed to detect the abnormal presence of double-stranded DNA (dsDNA) within the cytoplasm, a pathological hallmark of viral invasion or genomic instability. Upon binding cytoplasmic dsDNA in a sequence-independent manner, cGAS undergoes a conformational transition that activates its catalytic domain. This domain synthesizes the secondary messenger cyclic GMP-AMP (2′,3′-cGAMP) from ATP and guanosine triphosphate (GTP). Subsequently, cGAMP binds to STING, homodimerized on the ER membrane. This triggers STING translocation to the Golgi apparatus and recruits TANK-binding kinase 1 (TBK1), resulting in the subsequent phosphorylation and activation of interferon regulatory factor 3 (IRF3) and nuclear factor-kappa B (NF-κB) to orchestrate type I interferon (IFN-β) and pro-inflammatory cytokine expression. Importantly, experimental evidence demonstrates that photically triggered cGAS-STING activation via mitochondrial outer membrane disruption is predominantly achieved using bioengineered PDT and PTT nanoplatforms, rather than canonical antibody-IR700 NIR-PIT [8,76]. Crucially, the acute generation of ^1^O_2_ and hydroxyl radicals OH∙ during photodynamic nanotherapy disrupts the integrity of the mitochondrial respiratory chain and induces severe outer membrane permeabilization, leading to the direct leakage of mitochondrial dsDNA (mtDNA) into the cytoplasm [76]. Once in the cytosol, mislocalized mtDNA acts as a potent endogenous driver of cGAS; importantly, cGAS recognizes cytosolic dsDNA in a sequence-independent manner governed primarily by DNA duplex length and structural conformation rather than specific nucleotide motifs, driving 2:2 cGAS–dsDNA oligomerization, biomolecular phase separation, and catalytic 2′,3′-cGAMP synthesis [76]. Concurrently, if photically released mtDNA is discharged into the extracellular milieu and internalized into the endolysosomal compartment of infiltrating antigen-presenting cells, its evolutionary retention of unmethylated CpG motifs enables complementary engagement of endosomal Toll-like Receptor 9 (TLR9), thereby operating in tandem with the cytosolic cGAS-STING axis to amplify type I interferon expression and professional dendritic cell cross-presentation [76]. Furthermore, this nano-enabled activation has been strategically amplified using smart metal–organic framework (MOF) nanoconverters [76]. For instance, in the primary work establishing the cDZ@IP platform, polydopamine-coated zeolitic imidazolate frameworks (ZIF-8) co-loaded with ICG and topotecan dramatically intensified mitochondrial disruption [77]. Under near-infrared laser irradiation, ICG-mediated photothermal stress synergized with topotecan-induced nuclear/mitochondrial damage to provoke massive cytosolic mtDNA extrusion. Simultaneously, the acid-triggered degradation of the ZIF-8 matrix released free zinc ions ($Zn^{2+}$), which functioned as essential enzymatic co-factors that directly enhanced the binding affinity between cGAS and the leaked dsDNA, markedly amplifying downstream 2′,3′-cGAMP synthesis and STING signaling [77]. This photodynamic engagement of cGAS-STING represents a distinct immunogenic mechanism compared to conventional localized ablation. In comparative oncology models, the acute vascular destruction and rapid cell lysis mediated by vascular-targeted photodynamic therapy (VTP) triggers a significantly more robust engagement of the STING-regulated gene expression program than ionizing radiation therapy [3]. While radiotherapy-induced DNA damage is heavily dependent on cell cycle progress and often muted by double-strand break repair systems, VTP-induced organelle damage causes immediate, widespread cytosolic dsDNA leakage, driving a robust, systemic abscopal response mediated by peripheral expansion of CD8+ T-cell clones [3]. Additionally, incorporating oxygen-generating elements into photosensitive platforms, such as engineering outer membrane bacterial vesicles with catalases to decompose hydrogen peroxide (FeSR780@CAT@Mex-RS17), effectively relieves TME hypoxia, restoring the oxygen levels required for photodynamically induced ROS generation, mitochondrial lipid peroxidation, and subsequent mtDNA-mediated cGAS-STING engagement [78]. In parallel, multifunctional zinc-based targeted nanoagonists responsive to both the TME and NIR light have been engineered to synergistically amplify STING-dependent innate cascades, combining direct photothermal stress with sustained cytosolic pathway activation [77].

### 6.2. Co-Delivery of Synthetic STING Agonists (cGAMP, MSA-2, SR717) via Smart Nanoparticles

While the photic release of endogenous mtDNA represents a powerful intrinsic trigger, the immunosuppressive, adenosine-rich, and hypoxic TME often limits downstream STING signaling. This highlights the therapeutic necessity of co-delivering highly potent, synthetic STING agonists directly alongside PSs. Synthesized STING agonists can be categorized into cyclic dinucleotides (such as cGAMP) and synthetic non-nucleotide small molecules (such as MSA-2 and SR717). However, the systemic delivery of free agonists is severely hindered by rapid enzymatic degradation by ectonucleotide pyrophosphatase/phosphodiesterase 1 (ENPP1), poor cellular uptake due to their highly hydrophilic and negatively charged structures, and the risk of inducing systemic cytokine storms. To address these pharmacokinetic bottlenecks, researchers have developed advanced bioengineered nanomedicines that co-package STING agonists and photosensitive agents in single, spatiotemporally controlled carriers [79]. One highly effective strategy utilizes carrier-free nanomedicines composed entirely of API. For example, self-assembled carrier-free Ce6&SR717 nanoparticles combine the Ce6 with the non-nucleotide STING agonist SR717 via stable hydrophobic and electrostatic interactions [80]. Following local administration and tumor accumulation, NIR-induced Ce6 photodynamics spark intense ROS production and ICD. This localized stress coordinates with the synchronized intracellular release of SR717, triggering robust cGAS-STING pathway activation. This combined action stimulates a powerful, type I IFN-driven dendritic cell maturation cascade, leading to the recruitment and activation of cytotoxic T cells that induce a profound systemic abscopal response in contralateral, non-irradiated breast tumors [80]. Similarly, non-nucleotide small-molecule agonists like MSA-2 can be integrated into near-infrared II (NIR-II) responsive platforms. An NIR-II-immunostimulatory nanoplatform loaded with the PS IRM and MSA-2 achieves deep-tissue penetration, facilitating mild PTT combined with STING activation [81]. Preclinical models have validated that the resulting therapeutic efficacy and the complete regression of distant metastatic lesions are strictly dependent on the cGAS-STING axis, with therapeutic benefits entirely abolished in STING knockout (STING-/-) mouse models [81]. This highlights the mechanistic specificity of these combination therapies. In addition to non-nucleotide agonists, cyclic dinucleotides like cGAMP have been successfully delivered using multi-functional inorganic nanoplatforms. A novel selenium-doped gold nanostructure (Au-Se) designed for the co-delivery of cGAMP and acriflavine (a potent inhibitor of HIF-1α provides a highly coordinated therapeutic mechanism [82]. Under laser irradiation, the Au-Se platform induces mild localized hyperthermia, promoting the release of cGAMP to engage STING. Simultaneously, the released acriflavine successfully blocks HIF-1α translation, preventing the transcription of VEGF and the recruitment of immunosuppressive MDSCs. This dual-action platform effectively remodels the immunological landscape of the TME, facilitating cytotoxic CD8+ T-cell infiltration and inducing systemic immune responses in preclinical models [82].

### 6.3. Biomimetic Membrane-Camouflaged Vesicles and Scaffolds for Targeted STING Engagement

To further enhance tumor targeting, prolong blood circulation, and minimize off-target toxicities, biomimetic nanotechnology has emerged as a frontline strategy. This approach involves coating drug-loaded nanocarriers with cell membranes derived from platelets, macrophages, or cancer cells. For instance, biomimetic nanovesicles functionalized with Programmed Death-1 (PD-1) receptors (CDN@PM-ICG) are engineered by camouflaging ICG and cGAMP-loaded cores with PD-1-enriched macrophage membranes [83]. The surface-expressed PD-1 molecules bind specifically to PD-L1 overexpressed on cancer cells, achieving precise active targeting. Upon light activation, ICG induces localized ICD while the concurrent intracellular delivery of cGAMP drives robust cGAS-STING activation, preventing the immune evasion of hepatocellular carcinoma in vivo [83]. Similarly, homotypic tumor targeting can be achieved by utilizing cancer-cell-membrane coatings. Biomimetic STING nanoagonists wrapped in CRT-enriched fibrosarcoma membranes (GIP@CRTM) and co-loaded with cGAMP and ICG demonstrate an exceptional ability to accumulate in homotypic tumor beds [84]. This CRT-rich biomimetic coating serves as a potent adjuvant that accelerates antigen recognition by professional antigen-presenting cells. When activated by NIR light, the synchronized localized hyperthermia and cGAMP-mediated STING engagement trigger dendritic cell maturation and robustly expand tumor-specific cytotoxic CD8+ T lymphocytes, proving highly effective in neoadjuvant settings [84]. Beyond spherical nanovesicles, the integration of cGAS-STING activating elements into solid biomaterials has yielded promising results for post-operative care. In drug-resistant osteosarcoma, surgical resection of the primary bone tumor is frequently followed by local recurrence and metastatic dissemination. To address this clinical challenge, researchers have designed an immuno-chemotherapeutic bio-scaffold composed of biodegradable materials integrated with platinum (Pt(IV)) prodrugs and cucurbitacin B [85]. Sunder NIR stimulation, the scaffold triggers localized tumor cell death, releasing tumor-associated antigens and prompting cytoplasmic dsDNA accumulation. Concurrently, the scaffold releases cucurbitacin B, which acts as a powerful activator of dendritic cell antigen presentation and stimulates the cGAS-STING pathway. This localized, scaffold-driven activation significantly reduced residual drug-resistant osteosarcoma cell viability while promoting osteogenesis in experimental models (Table 6) [85].

## 7. Methodological Bottlenecks, Physiological Models, and Translational Benchmarks

### 7.1. The In Vitro-In Vivo Gap: Transitioning to 3D Microphysiological Systems

A critical methodological bottleneck in the development and validation of photoresponsive platforms is the historical reliance on traditional 2D monolayer cell cultures. While 2D assays offer high-throughput screening convenience, they fundamentally fail to replicate the complex physiological architecture and physical barriers of the TME. Specifically, 2D systems lack a three-dimensional ECM, multicellular heterogeneous cellular interactions, and realistic oxygen gradients (pO2). In the context of oxygen-dependent phototherapies, this creates highly artificial conditions where PSs are exposed to uniform, hyper-oxygenated states (normoxia in incubator settings, ~21% O_2_), leading to overestimations of singlet oxygen generation and phototoxical efficacy. Consequently, agents showing exceptional therapeutic indices in 2D frequently fail to produce meaningful tumor regression in vivo, where severe hypoxia and physical transport barriers restrict drug diffusion and photon penetration. To bridge this translational gap, current research has systematically transitioned to 3D microphysiological models, including multicellular tumor spheroids (MCTS) and patient-derived organoids (PDOs). These biomimetic models accurately recreate the dense, desmoplastic physical stroma, necrotic cores, and oxygen-depleted zones characteristic of solid human malignancies. Utilizing PDOs allows researchers to evaluate ‘photodynamic priming’ (PDP) strategies, demonstrating how sub-lethal optical stimulation can systematically deconstruct dense stromal barriers, lower IFP, and facilitate the deep penetration of subsequent systemically administered chemotherapeutics or immunotherapies. Furthermore, 3D spheroids are essential for evaluating advanced targeted platforms, such as co-packaged PARP inhibitor/PS assemblies and cascade-amplified nanoparticles, enabling realistic spatial mapping of drug transport, intracellular localization, and local immune activation markers under clinically relevant, oxygen-depleted conditions.

### 7.2. Clinical Gaps in Light Delivery, Dosimetry, and Safety

The clinical implementation of PIT presents significant technological and physiological challenges. The most prominent physical constraint is the tissue penetration depth of light. Even within the optical window of biological tissues (650–900 nm), light scattering and absorption by endogenous chromophores restrict effective photon delivery to a few centimeters. For superficial head and neck cancers, superficial illumination remains viable, but deep-seated, visceral, or thoracic malignancies require advanced endoscopic, interstitial, or wireless implantable light-delivery technologies. Designing flexible fiber-optic catheters, interstitial diffusing fibers, and biocompatible wireless LED arrays is essential to deliver uniform light dosimetry to complex, deep-tissue volumes while avoiding thermal injury to surrounding healthy structures. A parallel clinical hurdle is the standardization of immunological and physical dosimetry. Unlike conventional radiotherapy, which relies on well-defined ionizing radiation units, PIT requires a complex, multi-component dosimetry framework that accounts for APC plasma clearance, tumor receptor saturation, and tissue-specific light-fluence rates. Sub-optimal dosing can lead to incomplete cell ablation and promote compensatory survival pathways, while over-irradiation risks severe local toxicities. Clinical trials of EGFR-targeted antibody-dye conjugates (such as cetuximab sarotalocan) have identified specific adverse events, including intense local pain, transient laryngeal edema, and acute swelling within the light field, emphasizing the necessity of precise patient selection and specialized critical care management. Furthermore, local phototoxicity remains a substantial clinical risk. Intensive near-infrared irradiation can induce localized photodynamically driven vascular shutdown and tissue necrosis. In head and neck oncology, this can result in severe cutaneous and mucosal injuries, occasionally requiring advanced plastic reconstructive interventions (such as pedicled flap transfers) to manage tissue loss and promote vascular healing. This highlights the absolute clinical requirement for strict post-treatment photoprotection protocols, shielding patients from ambient light to prevent systemic skin injuries. Beyond these toxicological considerations, preclinical studies in syngeneic tumor models have robustly demonstrated that localized NIR-PIT can overcome primary and acquired resistance to systemic immune checkpoint blockade (anti-PD-1/anti-PD-L1), restoring cytotoxic T-cell functional activity and driving abscopal tumor regression upon re-challenge [51,54]. In the clinical setting, translational evidence exploring this paradigm is actively emerging: preliminary findings from early-phase clinical trials combining cetuximab sarotalocan (ASP-1929) with pembrolizumab in patients with recurrent or metastatic HNSCC previously progressing on or refractory to anti-PD-1 therapy have shown encouraging disease control rates and objective tumor shrinkage, suggesting potential microenvironmental resensitization to systemic checkpoint inhibition [7,86]. Nevertheless, bona fide, predictable systemic abscopal complete remissions in checkpoint-refractory cancer patients have not yet been definitively established in randomized phase 3 trials, underscoring the gap between preclinical promise and validated clinical efficacy [7,61]. In parallel, light-activated virus-like drug conjugates (such as belzupacap sarotalocan, Bel-sar) have demonstrated favorable safety profiles and localized tumor-infiltrating lymphocyte recruitment in early-phase clinical evaluations for choroidal melanoma and non-muscle-invasive bladder cancer, providing clinical proof-of-principle for light-directed immuno-oncology [87,88]. Beyond local tissue edema and pain, residual skin phototoxicity remains a prominent clinical side effect, particularly when using long-circulating PSs. Strict photoprotection protocols are mandatory to shield patients from ambient sunlight and prevent severe systemic cutaneous lesions during the clearance phase.

### 7.3. Critical Appraisal: Tumor-Type Dependence, Immunological Heterogeneity, and Abscopal Inconsistency

A balanced translational assessment requires recognizing the significant biological and physical hurdles that prevent the uniform clinical efficacy of photo-immunotherapy across diverse oncology indications:

Physical Delivery vs. Light Penetration Constraints: It is a physical misconception that NIR-PIT “circumvents” light penetration limits. While the use of 690 nm photons offers deeper penetration than ultraviolet or visible light, photon intensity decays exponentially due to hemoglobin and water absorption as well as Mie and Rayleigh tissue scattering [10]. Consequently, solid tumors larger than 1–2 cm or those located within visceral organs cannot be ablated via non-invasive surface irradiation, demanding CT- or ultrasound-guided placement of interstitial needle diffusers, which introduces clinical risks of hemorrhage, visceral perforation, and incomplete illumination margins [7].

Receptor Heterogeneity and Antigen Downregulation: Antibody-IR700 conjugates depend strictly on high and homogeneous expression of the target antigen (e.g., EGFR, CD44, HER2) on every viable tumor cell. In clinical solid tumors, target antigen expression displays marked spatial heterogeneity [86]. Cancer cell sub-clones lacking sufficient receptor density escape biophysical membrane rupture, providing a cellular reservoir for rapid local recurrence unless combinatorial systemic targeting is achieved [86].

Immunological Resistance and Abscopal Rarity: The clinical translation of light-induced abscopal effects faces steep hurdles [61]. The release of DAMPs and neoantigens following photic lysis is a necessary, but frequently insufficient, condition to overcome systemic tolerance [12]. If the systemic host repertoire lacks high-affinity TCR clones, or if secondary metastatic niches express dominant inhibitory pathways (such as IDO1, CD73, or dense TGF-β fibrotic barriers), distant lesions remain clinically refractory despite robust primary site destruction [67,70]. Acknowledging these biological constraints is essential to identify which patient sub-populations are most likely to benefit from combinatorial regimens.

Clinical Failures, Acute Local Complications, and Safety Controversies: Alongside its therapeutic promise, the clinical deployment of photoimmunotherapy has encountered tangible clinical setbacks and severe safety alerts. A prominent concern in advanced head and neck oncology is the risk of catastrophic arterial hemorrhage. In patients where recurrent tumors encase the internal or external carotid artery, the rapid, photically driven necrosis of perivascular tumor tissue can precipitate acute carotid blowout syndrome, requiring strict radiological pre-screening and excluding tumors with major vascular invasion from treatment protocols [7,89]. Furthermore, extensive photic cytolysis in anatomically confined areas (such as the pharynx, larynx, or floor of the mouth) frequently induces acute localized edema, carrying substantial risks of post-procedural airway compromise that often necessitates preventive tracheostomy or prolonged airway monitoring in intensive care [7,89]. From an immunological standpoint, it remains controversial whether the localized burst of inflammation can inadvertently induce systemic hyperprogression or trigger severe immune-related adverse events (irAEs) when combined with systemic immune checkpoint blockade in patients with pre-existing autoimmune dispositions [12,51]. Acknowledging these clinical hazards is essential to establish stringent safety guidelines and delineate realistic patient eligibility criteria.

### 7.4. Health Economics, Infrastructure Requirements, and Financial Feasibility

A critical yet frequently overlooked barrier to the widespread adoption of photo-immunotherapy is the substantial economic burden and infrastructure complexity associated with this drug-device combination modality.

Capital Equipment and Device Disposables: Unlike conventional systemic pharmacotherapies, NIR-PIT mandates specialized, dedicated capital infrastructure. Treatment requires specialized multi-channel laser diode systems (e.g., the BioBlade™ 690 nm laser console) capable of delivering precisely calibrated fluence rates across multiple optical channels simultaneously [7]. Furthermore, for deep-seated or bulky tumors, illumination necessitates single-use, sterile cylindrical diffusing optical fibers and interstitial needle catheters [7,10]. In complex head and neck interventions requiring 4 to 8 interstitial fibers per treatment session, the cumulative cost of single-use fiber-optic hardware represents a significant per-session expenditure that surpasses standard radiotherapy consumables [90].

Operating Theater and Interventional Radiology Demands: The interstitial placement of light-delivery fibers cannot be conducted at the bedside; it requires prolonged operating room time under general anesthesia, or specialized interventional radiology suites equipped with high-resolution computed tomography (CT) or ultrasound guidance to ensure millimeters-accurate fiber trajectory planning and prevent vascular puncture [7]. These clinical logistics heavily amplify the indirect procedural and institutional overhead costs.

Drug-Device Pricing and Reimbursement Realities: As a benchmark, the clinical approval of cetuximab sarotalocan (Akesux™) in Japan established a reimbursement price exceeding $35,000 to $40,000 USD per single treatment cycle (combining the biologic conjugate and specialized disposables), excluding hospital stay and operative theater costs [90]. Additionally, post-treatment photoprotection demands dedicated light-controlled hospital rooms and continuous monitoring for up to 48 h to prevent severe ambient-light-induced skin erythema [7].

Cost-Effectiveness in Multimodal Oncology: In recurrent or metastatic head and neck cancers, where alternative regimens (such as pembrolizumab monotherapy or the EXTREME chemotherapy-cetuximab regimen) already impose considerable financial toxicity, justifying the cost-effectiveness of photo-immunotherapy requires formal health-technology assessments (HTA) [91]. Demonstrating whether the higher upfront cost of NIR-PIT is offset by durable locoregional disease control, organ-preservation benefits (sparing extensive mutilating salvage surgery), and improved quality-adjusted life years (QALYs) will be decisive for regulatory approval and public reimbursement in Western healthcare systems [90,91].

## 8. Conclusions

Photoimmunotherapy represents a targeted multimodal strategy that integrates localized physical tumor debulking with the potential to stimulate systemic antitumor immunity. By leveraging an oxygen-independent biophysical membrane disruption mechanism, canonical antibody-IR700 NIR-PIT circumvents the microenvironmental hypoxia that traditionally impairs conventional Type II photodynamic therapy [9]. However, light penetration remains an immutable physical boundary: 690 nm photon fluence diminishes rapidly in biological tissues, establishing that NIR-PIT cannot overcome tissue depth constraints without the aid of invasive interstitial diffusers or operative illumination [10]. When successfully executed, photic cytolysis triggers the release of DAMPs and native tumor antigens, promoting dendritic cell recruitment in immunogenic contexts [11,20]. Nevertheless, this modality should not be viewed as an unconditional remedy for immunologically cold tumors: the systemic induction of abscopal regression observed in syngeneic rodent models is heavily dependent on intact antigen-processing machinery, sufficient baseline tumor immunogenicity, and the effective deconstruction of concurrent immunosuppressive stroma [12,61]. Beyond primary tumor cytolysis, the true translational power of PIT lies in its capacity to comprehensively reprogram the immunosuppressive TME. Through the selective photic ablation of key cellular shields, such as Tregs, PMN-MDSCs, and CAFs, PIT deconstructs dense desmoplastic stroma, reduces IFP, and induces the Super-Enhanced Permeability and Retention effect, thereby drastically enhancing the intratumoral delivery of systemic therapeutic agents. Concurrently, photically leaked mitochondrial DNA activates the cytosolic cGAS-STING pathway, driving type I interferon production and orchestrating dendritic cell cross-presentation. When seamlessly integrated with systemic immunotherapies, including ICB targeting PD-1, PD-L1, CTLA-4, LAG3, or TIGIT, as well as intratumoral cytokines and adoptive cell therapies, PIT can serve as an in situ vaccine capable of promoting abscopal regression of untreated distant lesions and inducing protective immunological memory in preclinical models. Looking toward future clinical translation, several critical methodological, photophysical, and regulatory challenges must be systematically addressed to fully realize the potential of this multimodal approach. Preclinical evaluation must continue transitioning away from static two-dimensional monolayer cultures toward standardized human three-dimensional microphysiological systems, such as MCTS and PDOs, which accurately reproduce high IFP, dense ECM barriers, and realistic hypoxic gradients. Simultaneously, expanding the application of photoimmunotherapy to deep-seated visceral malignancies requires the technological advancement and scale-up of second near-infrared window PSs, implantable wireless LED devices, flexible fiber-optic diffusers, and X-ray-activated radiodynamic MOF scintillators to overcome the physical limits of photon penetration. Furthermore, establishing standardized, multi-component dosimetry algorithms that account for antibody-photoabsorber plasma kinetics, tissue receptor saturation, and light fluence rates remains vital to ensure complete tumor ablation while avoiding thermal injury and localized adverse events. Ultimately, integrating spatial transcriptomics and liquid biopsy metrics will enable biomarker-guided patient selection, paving the way for prospective randomized controlled clinical trials that can definitively establish photoimmunotherapy as a cornerstone of modern multimodal oncology.

## Figures and Tables

**Table 1:** Mechanistic Demarcation of Light-Activated Oncological Modalities.

Modality	Targeting/Delivery Agent	Photophysical Trigger	Primary Cytotoxic Mechanism	Oxygen Dependence	Primary Immunological Driver
Classical Antibody-IR700 NIR-PIT (Cancer- or Immune-targeted)	Monoclonal antibody (e.g., cetuximab, panitumumab) conjugated to IRDye700DX	690 nm NIR light	Photochemical cleavage of axial ligands → hydrophobic aggregation → biophysical membrane rupture and osmotic burst	Oxygen-Independent	Immediate, synchronized release of native DAMPs and un-denatured tumor-associated antigens
Conventional PDT	Systemic or topical small-molecule photosensitizers (e.g., porphyrins, chlorins)	Vis/NIR light (400–700 nm)	Type I (electron transfer →$O_{2}^{∙-}$, OH∙) and Type II (energy transfer →O21) ROS generation	Strictly Oxygen-Dependent (compromised in hypoxia)	ROS-induced oxidative stress, ER stress, and secondary ICD
Photothermal Therapy (PTT)	Nanoparticles (Au, Prussian blue, CuS) or organic dyes (ICG)	NIR-I/NIR-II light (700–1100 nm)	Non-radiative optical absorption generating localized hyperthermia (43 °C), causing protein coagulative necrosis	Oxygen-Independent	Thermal tumor ablation accompanied by heat-shock protein (Hsp70/Hsp90) release
Nano-Photoimmunotherapy (Nano-PIT)	Biomimetic vesicles, MOFs, polymer micelles co-loaded with PSs, PTAs, or adjuvants	NIR light or ultrasound/X-ray stimuli	Combined Type I/II ROS, localized hyperthermia, and catalytic relief of hypoxia (e.g., nanozymes)	Variable (frequently engineered to bypass hypoxia)	Orchestrated DAMP release, STING activation, and targeted checkpoint/stromal deconstruction

**Table 2:** Comprehensive Analysis of Advanced Bioengineered Nano-PIT Platforms.

Platform Name	Active Ingredients/Components	Targeting and Stimuli Mechanism	Photochemical/Cytotoxic Mechanism	Immunological Outcome
G-IrC8 (Biomimetic Hybrid Vesicles)	Iridium(III) complex (IrC8) co-loaded into giant plasma membrane vesicles (GPMVs)	Homotypic self-recognition targeting derived from cancer cell membranes	Mitochondrial-localizing ROS generation under NIR light, causing immediate membrane lysis	Induction of classical necrotic ICD and subsequent dendritic cell (DC) maturation
Hybrid γδ-T Exosomes	Endogenous granzyme B, perforin, granulysin + loaded photosensitizers	Innate homing via CCR5 chemokine receptor towards tumor parenchyma	Synergistic protein toxicity and light-induced singlet oxygen (Type-II ROS)	Highly immunogenic necrosis, robust DC priming, expansion of CTL clones
Z/H@DFMNs (DNA-Nanoflower Microneedles)	Artificial DNA-zyme (G-quadruplex/hemin) + zinc phthalocyanine (ZnPc) + PD-L1 ASO	Transdermal application, tumor microenvironment H_2_O_2_ stimuli-responsive	Enzymatic O_2_ generation to alleviate tumor hypoxia, fueling ZnPc-mediated singlet oxygen production	Synergistic transdermal PDT-immunotherapy with PD-L1 gene silencer ASO blockade
EM@TCN (Hybrid Membrane Nanoplate)	Golgi-targeted AIE PS encapsulated in macrophage cell membrane hybrids	Organelle targeting (Golgi apparatus) via membrane hybrid molecules	Golgi apparatus swelling and immediate physical cell rupture under light	Coordinated induction of GSDMD-dependent pyroptosis and classical ICD
cypate@EXO-CXCL9 (Engineered Exosomes)	Chemokine CXCL9 + PS cypate within exosomal core	Receptor-mediated targeting; laser-responsive release of chemokine	Local photic ablation combined with passive/active drug extravasation	Active CXCL9-driven cytotoxic T lymphocyte recruitment; TAM M2-to-M1-like repolarization
ID-TEX (Homotypic Tumor Exosomes)	Endogenous IR808 + pH-responsive hydrazone-linked doxorubicin	Homotypic targeting to parent cancer cells; pH-responsive release	Sequential chemo-phototherapy (DNA intercalation and light-induced ROS)	Dual-targeted mechanical and chemical ICD induction in OSCC models

**Table 3:** Combinatorial Regimens of Local Phototherapy and Systemic Immunotherapy.

Local Phototherapy	Systemic Immunotherapy	Combinatorial Mechanism and Therapeutic Outcome
NIR-PIT (cetuximab-IR700)	Systemic anti-PD-1 antibody	Rapid selective necrosis and neoantigen shedding paired with anti-exhaustion checkpoint blockade, preventing tumor recurrence.
NIR-PIT (anti-PD-L1-IR700)	Pegylated Interferon-gamma (IFN-g)	Pre-treatment upregulates PD-L1 density, enhancing susceptibility to photic cytolysis and increasing CD8+ CTLs infiltration.
Repeated PDT (porphyrin-lipoproteins)	Systemic anti-PD-1 antibody	Sustained innate and adaptive activation (IL-6 increase), amplifying non-irradiated abscopal tumor regression under combination therapy.
Dual EGFR- and CTLA-4-targeted PIT	In situ Treg depletion via anti-CTLA-4-IR700	Functional cooperativity: simultaneous primary tumor debulking and removal of local Treg brakes, favorably shifting intratumoral CD8+/Treg ratio.
CD29-targeted NIR-PIT	Systemic anti-CTLA-4 antibody	Sparing of bystander tumor-infiltrating lymphocytes combined with systemic checkpoint inhibition, eliciting tumor regression in melanoma.
Ce6 PDT (triggers PANoptosis)	LAG3 blockade and STING agonist	nduces inflammatory PANoptosis, suppresses systemic MDSCs, and enhances recruitment of cDC2 and cytotoxic CD8+ T cells.
CD25-targeted NIR-PIT (Treg depletion)	Intratumoral Interleukin-15 (IL-15)	Cooperative local immunosuppression reversal, preventing CD8+ exhaustion and overcoming primary resistance to systemic anti-PD-1.
EGFR-targeted NIR-PIT	Intratumoral Interleukin-15 (IL-15)	Additive immunotherapeutic stimulation: increases local NK and CD8+ effector cell density, inhibiting spontaneous lung metastases.
Liposomal ICG PTT (vaccine)	Flagellin (FlaB) adjuvant and anti-PD-1	Photothermal antigen release combined with TLR5 dendritic cell agonism, achieving 90% systemic regression when co-administered with anti-PD-1.
Gold Nanorod-mediated PTT	Intratumoral IL-2 or anti-PD-1	Favorable modulation of CD8+/Treg ratios in non-irradiated distant metastatic sites, establishing systemic anti-tumor immunity.
Ag@CuS-TPP@HA NIR-II PTT	Systemic anti-PD-L1 antibody	Deep-tissue NIR-II photothermal ROS generation paired with PD-L1 checkpoint blockade, suppressing metastatic pulmonary burden.
Conventional PDT	Polymer nanoparticles with CpG/peptides	Accelerates the cancer-immunity cycle by co-delivering CpG TLR9-agonists and tumor-specific peptides to antigen-presenting cells.
Prussian Blue Nanoparticle PTT	Ex vivo engineered T-cell expansion	PTT-ablated tumor cells act as pristine antigen sources for ex vivo dendritic cell priming, generating cytotoxic T memory cells.
GD2-targeted NIR-PIT	Adoptive Cell Therapy (NK/NKT)	Ablates tumor cells and remodels extracellular stroma, facilitating homing and infiltration of adoptively transferred NK/NKT cells.

**Table 4:** Translational and Developmental Spectrum of Photo-Immunotherapy Modalities and Combinatorial Regimens.

Developmental Tier	Target/Combination	Phototherapeutic Modality and Platform	Target Indication/Model	Key Evidence and Clinical/Translational Milestone
Approved in Clinical Practice	EGFR (monotherapy)	Classical NIR-PIT: Cetuximab sarotalocan (ASP-1929) + 690 nm laser (BioBlade™)	Locally recurrent, unresectable head and neck squamous cell carcinoma (HNSCC)	Approved by PMDA (Japan); produces targeted biophysical membrane rupture and rapid tumor cytolysis in clinical practice
Active Clinical Trials	EGFR + Systemic Anti-PD-1	Cetuximab sarotalocan (ASP-1929) combined with anti-PD-1 (pembrolizumab)	Recurrent/metastatic HNSCC and advanced solid malignancies	Phase 1b/2 and Phase 3 clinical evaluation; demonstrates targeted debulking and reversal of resistance to anti-PD-1 checkpoint blockade
Active Clinical Trials	Heparan sulfate proteoglycans	Bel-sar (AU-011, light-activated virus-like drug conjugate)	Primary indeterminate lesions/choroidal melanoma; non-muscle-invasive bladder cancer	Early-phase clinical trials demonstrating favorable safety, localized tumor destruction, and immune cell infiltration
Preclinical Proof-of-Concept	EGFR + Intratumoral Treg Depletion (CTLA-4)	Dual cancer- and immune-targeted NIR-PIT (cetuximab-IR700 + anti-CTLA-4-IR700)	Syngeneic murine tumor models	Synergistic primary tumor ablation and removal of local Treg-mediated immunosuppression; bilateral tumor regression
Preclinical Proof-of-Concept	CD25-targeted NIR-PIT + Intratumoral IL-15	Anti-CD25-IR700 conjugate with local IL-15 cytokine administration	Murine immunocompetent cancer models	Overcomes primary resistance to systemic anti-PD-1; activates local NK and effector CD8+ T cells
Preclinical Proof-of-Concept	Photodynamic PANoptosis + LAG3 Blockade	Ce6 derivative PDT with LAG3 checkpoint inhibitor	Murine cutaneous squamous cell carcinoma	Reprograms myeloid cells, recruits type-2 conventional dendritic cells (cDC2) and CD8+ CTLs
Preclinical Proof-of-Concept	cGAS-STING Activation + ICB	Self-assembled carrier-free nanoassemblies (Ce6&SR717)/Zinc MOFs (cDZ@IP)	Murine breast cancer and syngeneic solid tumors	Nanoparticle-mediated STING agonism triggering type I IFN and abscopal regression of non-irradiated contralateral tumors
Preclinical Proof-of-Concept	In Situ Cancer Vaccination	Liposomal ICG PTT with flagellin adjuvant (FlaB) + anti-PD-1	Orthotopic syngeneic murine tumor models	TLR5 stimulation on DCs; triggers up to 90% systemic regression and long-term protection against rechallenge
Preclinical Proof-of-Concept	Adoptive Cell Therapy (ACT) Priming	GD2-targeted NIR-PIT/Prussian blue nanoparticle photothermal antigen priming	Preclinical neuroblastoma and engineered cell transfer models	Stroma/ECM deconstruction facilitating adoptive NK/NKT infiltration; ex vivo expansion of memory T cells

**Table 5:** Strategies for Overcoming Immunosuppression in the TME via Photic and Nanotechnology Platforms.

Barrier Type	Target/Platform	Molecular Mechanism	Key Immunological Outcome
Cellular Shield	Tregs (CD25)/Ly6G (PMN-MDSCs)	Antibody-IR700 binding followed by 690 nm NIR light; rapid biophysical membrane rupture	Localized depletion of suppressive populations; CD8+ T and NK cell reactivation in treated TME
Metabolic Pathway	COX-2/PGE2/IDO1 (DHU-CBA2, NLG919)	Enzymatic inhibition of prostaglandin synthesis and tryptophan-to-kynurenine conversion	Prevents T-cell anergy; suppresses Treg differentiation; synergizes with anti-PD-L1 checkpoint blockade
Metabolic Pathway	CD73/Adenosine (AptEM@CBA, CRISPR/Cas9)	Enzymatic CD73 blockade (AMPCP) or Cas9-mediated CD73 gene knockout	Prevents conversion of ATP to immunosuppressive adenosine; maintains CTL activity
Stromal Barrier	FAP/PDPN (CAFs) (Anti-FAP-IR700)	Photic ablation of CAFs; Losartan collagen synthesis blockade; ETM@PT nanoparticles	Reduces collagen I/hyaluronic acid; lowers interstitial fluid pressure (IFP); restores drug perfusion
Hypoxia Circuit	HIF-1α/HSP70/PD-L1 (MLT, AuNH-2-Ab)	Oxygen generation by nanozymes; melatonin-mediated HIF-1 α suppression; pH/thermal PD-L1 release	Reverses tumor thermo-resistance; blocks TAM-mediated crosstalk; enhances NK cell surveillance

**Table 6:** Specialized Photic Platforms for cGAS-STING and Innate Immune Activation.

Platform Name and Composition	STING Stimulating Agent	Photic Co-Activation and Mechanism	Key Immunological Outcome
cDZ@IP (ZIF-8 + ICG + Topotecan) [40]	Endogenous mtDNA + Zinc co-factor amplification	ICG-mediated PTT + Topotecan DNA damage causing massive mtDNA cytosolic leakage	Enhanced cGAS-mtDNA binding affinity, massive IFN-β expression, and DC cross-priming
Ce6&SR717 Carrier-Free Nanoassembly [80]	Synthetic non-nucleotide agonist SR717	Ce6-induced photodynamic ROS generation triggering ICD	Synchronized DAMP release + STING agonism; robust abscopal regression of distant tumors
Au-Se Nanoplatform + Acriflavine [82]	Cyclic dinucleotide cGAMP	Au-mediated localized hyperthermia triggering release + acriflavine HIF-1a inhibition	Dramatically reduced VEGF transcription, suppressed MDSCs, and expanded CTL CD8+ infiltration
CDN@PM-ICG PD-1 Biomimetic Vesicles [83]	Cyclic dinucleotide cGAMP	PD-1 active targeting to PD-L1 + ICG-mediated local PDT	Targeted tumor ICD induction combined with targeted cGAMP release, bypassing ENPP1 degradation
FeSR780@CAT@Mex-RS17 Catalase-OMVs [78]	Endogenous mtDNA + ROS-induced ferroptosis	Catalase decomposition of H_2_O_2_ to generate O_2_, relieving hypoxia and driving PDT	Alleviated tumor hypoxia, restoring oxygen levels required for robust mtDNA-mediated cGAS activation
TME/NIR Dual-Responsive Zinc Nanoagonist [77]	Synthetic STING agnonist + Zn^2+^ co-delivery	Near-infrared-triggered mild phototermal activation and targeted microenvironmental release	Synergistic enzymatic cGAS-STING amplification, systemic CTL infiltration, and eradication of distant metastases

## Data Availability

No new data were created or analyzed in this study. Data sharing is not applicable to this article.

## Abbreviations

The following abbreviations are used in this manuscript:

PDT	Photodynamic Therapy
NIR-PIT	Near-Infrared Photoimmunotherapy
PTT	Photothermal Therapy
VTP	Vascular-Targeted Photodynamic Therapy
ICD	Immunogenic Cell Death
ICB	Immune Checkpoint Blockade
ACT	Adoptive Cell Therapy
PDP	Photodynamic Priming
ROS	Reactive Oxygen Species
TME	Tumor Microenvironment
DAMPs	Damage-Associated Molecular Patterns
ECM	Extracellular Matrix
ER	Endoplasmic Reticulum
eIF2α	Eukaryotic Translation Initiation Factor 2 Alpha
DCs/iDCs/mDCs	Dendritic Cells/immature DCs/mature DCs
CTLs	Cytotoxic T Lymphocytes
Tregs	Regulatory T Cells
MDSCs/PMN-MDSCs	Myeloid-Derived Suppressor Cells/Polymorphonuclear MDSCs
TAMs	Tumor-Associated Macrophages
CAFs	Cancer-Associated Fibroblasts
NK	Natural Killer
TDLNs	Tumor-Draining Lymph Nodes
CD	Cluster of Differentiation
EGFR	Epidermal Growth Factor Receptor
LAG3	Lymphocyte-Activation Gene 3
HIF-1α	Hypoxia-Inducible Factor 1-alpha
VEGF	Vascular Endothelial Growth Factor
CRT	Calreticulin
ATP	Adenosine Triphosphate
HMGB1	High Mobility Group Box 1
Hsp	Heat Shock Proteins
GSDM	Gasdermin
TNF-α	Tumor Necrosis Factor alpha
TBK1	TANK-Binding Kinase 1
IRF3	Interferon Regulatory Factor 3
IDO1	Indoleamine 2,3-Dioxygenase 1
FAP	Fibroblast Activation Protein
PDPN	Podoplanin
MMP9	Matrix Metalloproteinase-9
ENPP1	Ectonucleotide Pyrophosphatase/Phosphodiesterase 1
PSs	Photosensitizers
APC	Antibody-Photoabsorber Conjugate
API	Active Pharmaceutical Ingredient
GPMVs	Giant Plasma Membrane Vesicles
EVs	Extracellular Vesicles
Ce6	Chlorin e6
AIE	Aggregation-Induced Emission
MOF	Metal–Organic Framework
MCTS	Multicellular Tumor Spheroids
PDOs	Patient-Derived Organoids
OSCC	Oral Squamous Cell Carcinoma
PDAC	Pancreatic Ductal Adenocarcinoma
TNBC	Triple-Negative Breast Cancer
